# Effect of Metformin on the Risk of Post-COVID-19 Condition Among Individuals With Overweight or Obese: A Population-based Retrospective Cohort Study

**DOI:** 10.1093/cid/ciaf429

**Published:** 2025-09-01

**Authors:** Ubonphan Chaichana, Kenneth K C Man, Chengsheng Ju, Janine Makaronidis, Li Wei

**Affiliations:** Research Department of Practice and Policy, UCL School of Pharmacy, London, United Kingdom; Research Department of Practice and Policy, UCL School of Pharmacy, London, United Kingdom; Laboratory of Data Discovery for Health (D24H), Hong Kong Special Administrative Region, China; Centre for Medicines Optimisation Research and Education, University College London Hospitals National Health Service (NHS) Foundation Trust, London, United Kingdom; Centre for Safe Medication Practice and Research, Department of Pharmacology and Pharmacy, Li Ka Shing Faculty of Medicine, The University of Hong Kong, Hong Kong Special Administrative Region, China; Research Department of Practice and Policy, UCL School of Pharmacy, London, United Kingdom; Institute of Cardiovascular Science, University College London, London, United Kingdom; Centre for Obesity Research, Rayne Institute, Department of Medicine, University College London, London, United Kingdom; National Institute of Health and Care Research, University College London Hospitals Biomedical Research Centre, London, United Kingdom; Department of Diabetes and Metabolism, Royal London Hospital, London, United Kingdom; Research Department of Practice and Policy, UCL School of Pharmacy, London, United Kingdom; Laboratory of Data Discovery for Health (D24H), Hong Kong Special Administrative Region, China; Centre for Medicines Optimisation Research and Education, University College London Hospitals National Health Service (NHS) Foundation Trust, London, United Kingdom

**Keywords:** metformin, Post COVID-19 condition, long covid, COVID-19 preventive treatment

## Abstract

**Background:**

A subgroup analysis of the COVID-OUT trial's long-term outcome found that starting metformin within 3 days of coronavirus disease 2019 (COVID-19) diagnosis reduced post–COVID-19 condition (PCC) incidence by 63% in overweight or obese individuals. However, its generalizability remains uncertain.

**Objectives:**

To evaluate the effectiveness of metformin in preventing PCC in adults with overweight or obesity who had a recent COVID-19 infection.

**Design:**

A retrospective cohort study using a sequential target trial emulation framework.

**Data Sources:**

The United Kingdom primary care data from the Clinical Practice Research Datalink Aurum database from March 2020 to July 2023.

**Participants:**

Adults with overweight or obesity (body mass index ≥ 25 kg/m²) and a record of severe acute respiratory syndrome coronavirus 2 infection were included. Exclusions included metformin use in the prior year or metformin contraindications.

**Measurements:**

The outcome was PCC, defined by a PCC diagnostic code or at least 1 World Health Organization–listed symptoms between 90 and 365 days after diagnosis, with no prior history of the symptom within 180 days before infection. The pooled hazard ratio and risk difference for the incidence of PCC were adjust for baseline characteristics.

**Results:**

Among 624 308 patients, 2976 initiated metformin within 90 days of COVID-19 diagnosis. The 1-year risk difference for PCC in the intention-to-treat analysis was −12.58% (hazard ratio 0.36; 95% CI, 0.32–0.41), with consistent results in subgroup analyses.

**Limitations:**

Findings may not apply to individuals with a normal body mass index.

**Conclusions:**

Early metformin treatment in overweight or obese individuals may reduce PCC risk. Further research is needed to confirm causality and clarify metformin's role in PCC management.

## Teaser

Early metformin treatment in overweight or obese adults with recent coronavirus disease 2019 infection significantly reduces the risk of developing post–coronavirus disease-19 condition. This retrospective cohort study supports metformin's potential role in post–COVID-19 condition prevention, warranting further research to confirm causality.

**(See the Editorial Commentary by Bramante and Boulware on pages e433–5.)**

Post-coronavirus disease (COVID-19) condition (PCC), or long COVID, is a complex condition with >200 identified symptoms affecting multiple organ systems [1]. Common symptoms include brain fog, fatigue, persistent cough, shortness of breath, and insomnia [2–5]. The UK's National Institute for Health and Care Excellence defines PCC as symptoms persisting for at least 4 weeks after acute COVID-19 [6], whereas the World Health Organization (WHO) defines it as lasting at least 2 months, usually beginning 3 months postinfection [7]. As of March 2023, 1.9 million people in the United Kingdom (2.9% of the population) reported experiencing PCC [8]. Despite its prevalence, the long-term health impacts and treatment options remain unclear.

Metformin, a first-line treatment for type 2 diabetes (T2DM), lowers blood glucose by reducing gluconeogenesis and increasing peripheral glucose utilization and sensitivity [9, 10]. It exerts its effects primarily by activating adenosine monophosphate-activated protein kinase (AMPK), which enhances insulin sensitivity, inhibits hepatic glucose production, and promotes glucose uptake in peripheral tissues. Beyond its glucose-lowering effects, metformin has anti-inflammatory properties, suppressing the expression of interleukin-1β, interleukin-6, TNFα, and adipokines [11]. These properties contribute to its protective effects against conditions such as cardiovascular disease, chronic kidney disease, and polycystic ovary syndrome [10, 12–14].

During the severe acute respiratory syndrome coronavirus 2 (SARS-CoV-2) pandemic, research has suggested that metformin may have antiviral properties by targeting interactions between SARS-CoV-2 proteins and human proteins [15, 16]. It may also inhibit infection by interacting with the human angiotensin-converting enzyme 2 through AMPK [17]. Despite these potential mechanisms, clinical evidence remains inconsistent. Some studies indicate that metformin use in COVID-19 patients with T2DM is associated with reduced mortality [18–20], particularly among women with T2DM or obesity [21].

Metformin's anti-inflammatory properties may help protect COVID-19 patients from developing PCC, though further research is needed to confirm its potential benefits [22]. Many ongoing studies worldwide are investigating treatments for PCC. A secondary analysis of the COVID-OUT trial showed promising results, indicating that when metformin was started within 3 days of a COVID-19 diagnosis and continued for 14 days, it reduced the incidence of PCC by 63% compared to a placebo [23]. However, these findings were limited to individuals aged 30 to 85 years, and PCC was a secondary outcome in the trial. Further research is necessary to determine whether metformin's preventive effects apply to a broader population and different PCC definitions, ensuring real-world applicability.

To address this knowledge gap, we conducted a study aiming to evaluate the effectiveness of metformin initiation post–COVID-19 infection on the development of PCC in individuals with overweight or obesity with regardless of their diabetic or glycemic status.

## METHODS

In this population-based cohort study, we used the Clinical Practice Research Datalink (CPRD) Aurum, a UK primary care database representing the population in England. CPRD Aurum includes demographic data, diagnoses, prescriptions, vaccinations, laboratory tests, and specialist referrals [24]. As of March 2024, it covers 1784 general practices (GPs), representing 24.15% of the population in England [25]. We linked Hospital Episode Statistics secondary care datasets and Office for National Statistics for mortality data [24].

### Study Design and Eligibility Criteria

We conducted a retrospective cohort study using a target trial emulation framework to compare the effect of metformin versus no metformin on developing PCC in individuals with overweight or obesity after SARS-CoV-2 infection. The specification and emulation of the target trial are presented in Supplementary Table 1. The baseline (T_0_) was set at the date of diagnosis for SARS-CoV-2 infection for trial 1, 30 days postdiagnosis for trial 2, and 60 days postdiagnosis for trial 3. The study included adults (≥18 years) with a body mass index (BMI) ≥ 25 kg/m² and a confirmed SARS-CoV-2 infection between March 2020 and March 2023, excluding those with less than 1 year of GP history, prior use of metformin or other glucose-lowering drugs within 1 year before the index date, history of nirmatrelvir use, or contraindications to metformin (hepatic or renal impairment or lactic acidosis). The study did not assess glycemic status because of the inclusion of individuals without diabetes.

### Treatment Strategies

In each emulated trial, we compared the treatment strategies of initiation and continued use of oral metformin treatment (defined as ≤120 days of metformin prescription gap), regardless of their diabetic status, at any dose within 30 days from study entry versus no metformin treatment during the entire follow-up time.

### Study Outcomes and Follow-up

The outcome PCC was defined as having a PCC diagnostic code using SNOMED-CT or International Classification of Diseases, 10th revision, Clinical Modification, codes in CPRD Aurum or having at least one of 25 WHO-listed symptoms during visits to a GP between 90 and 365 days after the COVID-19 diagnosis, with no history of that symptom 180 days before SARS-CoV-2 infection [26]. PCC diagnostic codes and 25 WHO-listed symptoms are presented in Supplementary Method 1. All patients were followed up from T_0_ until an occurrence of PCC, death, transfer out of the current practice, or 1 year after the date of the first record of SARs-CoV-2 infection diagnosis, whichever occurred first.

### Covariates

The included covariates were factors that may affect PCC symptoms and the use of metformin. We developed the covariate list based on previous published observational studies investigating the characteristics of PCC [27].

Covariates included age, gender, baseline BMI in categories, ethnicity, index of multiple deprivation quintiles, number of GP contact in the past year, COVID-19 vaccination status, smoking status, alcohol use status, SARS-CoV-2 dominant variant period, and recent drug prescriptions (within 180 days before T_0_). We used the measure that is closest to the study index date for BMI, socioeconomic status, smoking status, and alcohol consumption status. Common comorbidities that have similar symptoms of PCC were also assessed and defined as a record of disease at any time before the T_0_ point using the relevant read codes.

### Statistical Analysis

We emulated the sequential target trial protocols using observational data, classifying individuals into 2 groups based on their metformin prescription records. The study design diagram and an illustration of sequential trial emulation study for metformin treatment are presented in Supplementary Figures 1 and 2. The estimated cumulative incidence of PCC was compared between metformin users and nonusers using the Cox proportional hazard model to estimate hazard ratios (HRs) and risk differences (RDs).

To estimate the causal effects, both the observational analogue of the intention-to-treat (ITT) effect of initiating the treatment strategies and the per-protocol effect of adhering to the initiated treatment strategies.

For the ITT analysis, we employed weighted Cox proportional hazard models with an indicator for assigned treatment strategies, adjusting for baseline covariates using propensity-score fine stratification. Sequential trial emulation was used to enhance statistical efficiency, allowing us to estimate HRs and compare PCC event rates between metformin users and nonusers across the emulated trials.

In the per-protocol analysis, individuals in the metformin group were censored if they discontinued metformin or did not refill their prescription for >120 days. Similarly, individuals in the non-metformin group were censored on initiating metformin. However, individuals who discontinued metformin because of a contraindication were not censored. Pooled HR and RD from 3 emulated trials were obtained via individual-level meta-analysis with 500 bootstrap samples for 95% confidence interval (CI).

Baseline characteristics were presented as mean (standard deviation) for continuous variables and as number (%) for categorical variables. Standardized mean difference (SMD) was used to evaluate the difference in patient characteristics between 2 treatment groups before and after weighting. An SMD <0.2 is considered of good balance between 2 groups. A 2-sided *P* < .05 is considered as statistically significant.

### Secondary Analyses

Subgroup analyses were conducted on a subset of individuals based on age, BMI, gender, diabetes status, and the dominant SARS-CoV-2 variant at the time of COVID-19 diagnosis.

To ensure the robustness of our findings, we performed sensitivity analyses. First, we conducted a complete case analysis by excluding observations with missing data on smoking status, alcohol consumption, ethnicity, COVID-19 vaccination status, and index of multiple deprivation quintiles. This allowed us to assess the potential impact of missing data on our primary results.

Second, to evaluate the influence of unmeasured confounding, we performed a negative control outcome analysis using cancer diagnoses occurring 90–365 days after COVID-19 diagnosis as the outcome. A recent systematic review and meta-analysis of randomized controlled trials found that metformin did not significantly reduce cancer incidence in individuals with overweight/obesity, prediabetes, or diabetes [28], aligning with our study population characteristics. Additionally, a previous target trial emulation study reported no association between metformin use and reduced cancer risk [29]. Given the lack of strong evidence linking metformin to cancer incidence, we selected cancer as a negative control outcome to detect potential biases. Because metformin is unlikely to influence cancer development within the study period, any observed effects on PCC can be more confidently attributed to metformin rather than residual confounding or metabolic effects.

Additionally, we replicated the study using a traditional cohort design, initiating follow-up at 90 days after SARS-CoV-2 diagnosis to validate our results. Our study adheres to the Strengthening the Reporting of Observational Studies in Epidemiology (STROBE) guidelines for cohort studies [30]. All statistical analyses were performed using SAS version 9.4 (SAS Institute Inc., Cary, North Carolina).

## RESULTS

**Figure 1.**
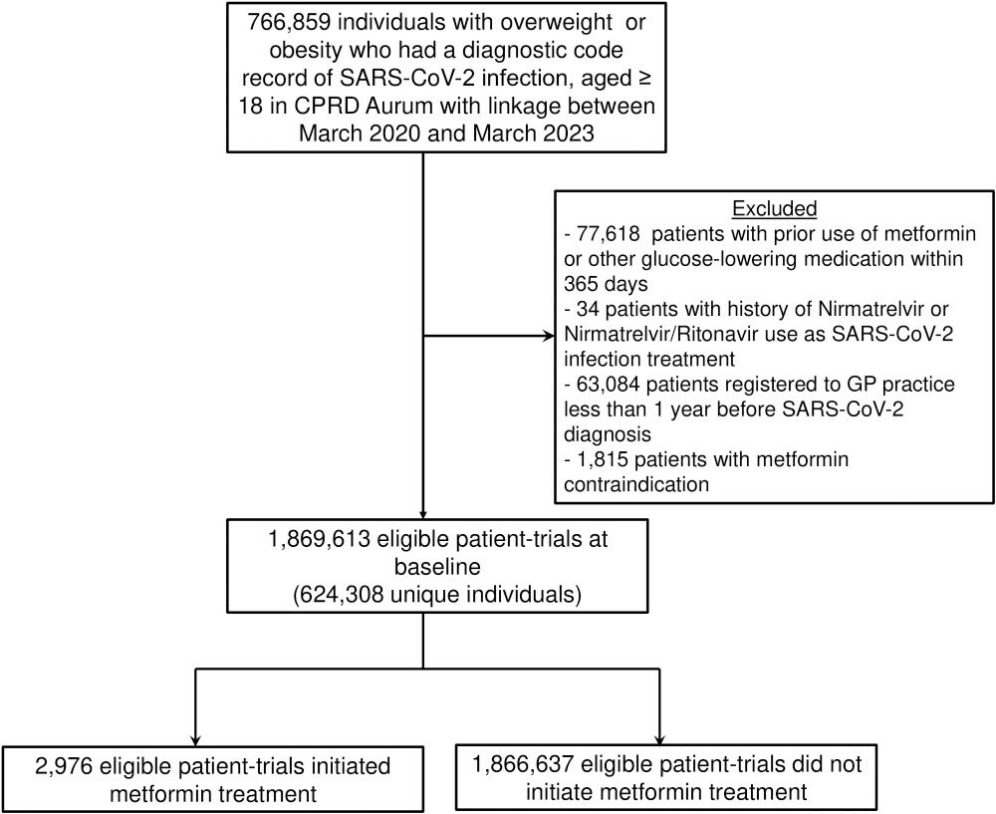
Flow chart of patient cohort selection. Abbreviations: CPRD, Clinical Practice Research Datalink; SARS-CoV-2, severe acute respiratory syndrome coronavirus 2.

A total of 766 859 overweight or obese patients with a record of SARs-CoV-2 infection were identified from the database during the study period. Of these, 624 308 patients met the eligibility criteria and were included in the analysis. There were 2976 patient-initiated metformin within 3 months after COVID-19 diagnosis date in this study. The mean age of this study cohort was 49.64 (15.38) years. Figure 1 shows a flowchart of patient selection for the three trials. Baseline characteristics of the individuals eligible for the emulated trials are shown in Table 1. All measured covariates were well balanced in all 3 emulated trials after applying propensity-score fine stratification weighting, with SMD <0.2; see Supplementary Tables 2–4 and Supplementary Figures 3–5.

**Table 1.** Baseline Characteristics of Eligible Individuals in the 3 Emulating Target Trials of Metformin Therapy and PCC Events Using Linked Electronic Health Records From Clinical Practice Research Datalink and Hospital Episode Statistics, 2020–2023

Characteristics^a^	Metformin Initiators (n = 2976)	Noninitiators (n = 1 866 637)
**Age (years), mean (SD)**	55.1 (15.8)	49.6 (15.4)
**BMI, kg/m** ^2^ **, n (%)**		
Overweight (25–29.9)	1028 (34.5)	1 065 557 (57.1)
Obesity class I (30–34.9)	913 (30.7)	488 526 (26.2)
Obesity class II (35–39.9)	584 (19.6)	192 650 (10.3)
Obesity class III (≥40)	451 (15.2)	119 904 (6.4)
**Sex, n (%)**		
Male	1405 (47.2)	729 014 (39.0)
Female	1571 (52.8)	1 137 623 (61.0)
**Ethnicity, n (%)**	…	**…**
White British	2199 (73.9)	1 492 086 (79.9)
Asian	290 (9.7)	107 564 (5.8)
Black	133 (4.5)	53 083 (2.8)
Others	112 (3.8)	58 671 (3.1)
Mixed	28 (0.9)	18 673 (1.0)
Missing	214 (7.2)	136 560 (7.3)
**Smoking status, n (%)**	…	**…**
Nonsmoker	1503 (53.2)	1 099 213 (58.9)
Ex-smoker	864 (29.0)	463 036 (24.8)
Current smoker	254 (8.5)	148 157 (7.9)
Missing	275 (9.2)	156 231 (8.4)
**Alcohol consumption status, n (%)**		
Current drinker	2608 (87.6)	1 557 280 (83.4)
Nondrinker	116 (3.9)	54 466 (2.9)
Ex-drinker	3 (0.1)	695 (0.1)
Missing	249 (8.4)	254 196 (13.6)
**GP contact in the past year, n (%)**		
0 time	1 (0.0)	1105 (0.1)
1–11 times	414 (13.9)	496 031 (26.6)
12–18 times	543 (18.3)	442 122 (23.7)
19–29 times	778 (26.1)	463 222 (24.8)
>29 times	1240 (41.7)	464 157 (24.9)
**Index of multiple deprivation, n (%)**		
1 (least deprived)	338 (11.4)	243 887 (13.1)
2	358 (12.0)	254 992 (13.7)
3	546 (18.4)	334 368 (17.9)
4	610 (20.5)	351 709 (18.8)
5 (most deprived)	677 (22.8)	396 131 (21.2)
Missing	447 (15.0)	285 550 (15.3)
**Comorbidities, n (%)**		
COPD	163 (5.5)	50 198 (2.7)
Asthma	708 (23.8)	433 728 (23.2)
Fibromyalgia	86 (2.9)	38 424 (2.1)
Anxiety	778 (26.1)	477 169 (25.6)
Depression	441 (14.8)	232 352 (12.5)
Migraine	410 (13.8)	271 355 (14.5)
Arrhythmia	341 (11.5)	75 223 (4.0)
Osteoporosis	629 (21.1)	266 504 (14.3)
Fragility fracture	35 (1.2)	17 057 (0.9)
Eczema	681 (22.9)	440 286 (23.6)
Type 2 diabetes	1543 (51.9)	47 043 (2.5)
Hypertension	1187 (39.9)	405 253 (21.7)
Liver disease	23 (0.8)	8775 (0.5)
Dementia	31 (1.0)	10 288 (0.6)
Chronic kidney disease	245 (8.2)	76 841 (4.1)
Stroke	187 (6.3)	49 247 (2.6)
Coronary heart disease	286 (9.6)	68 915 (3.7)
Heart failure	374 (12.6)	28 485 (1.5)
PCI	76 (2.6)	14 387 (0.8)
Rheumatoid	53 (1.8)	26 172 (1.4)
Peptic ulcer	81 (2.7)	28 599 (1.5)
Cancer	202 (6.8)	85 249 (4.6)
Myocardial infarction	237 (8.0)	36 047 (1.9)
**Use of medication in the past 6 mo, n (%)**		
HRT^b^ (% of women)	80 (5.1)	138 299 (12.2)
Antipsychotic	115 (3.9)	49 958 (2.7)
Antidepressants	804 (27.0)	429 695 (23.0)
CNS drugs use^c^	6 (0.2)	3777 (0.2)
CVD drugs use^d^	1632 (54.8)	571 242 (30.6)
NSAIDs	1134 (38.1)	491 315 (26.3)
Opioid drug	1323 (44.5)	456 500 (24.5)
**COVID-19 vaccination status, n (%)**		
Unvaccinated	59 (1.9)	18 848 (1.0)
1 dose	293 (9.9)	233 456 (12.5)
2 doses	967 (32.5)	729 708 (39.1)
3 doses or more	1071 (36.0)	616 562 (33.0)
Missing	586 (19.7)	268 063 (14.4)
**Dominant SARs-CoV-2 variants at the COVID-19 diagnosis date, n (%)**		
Pre-Alpha and Alpha period (≤17/05/2021)	468 (15.7)	174 131 (9.3)
Delta period (18/05/2021–13/12/2021)	682 (22.9)	545 536 (29.2)
Omicron period (14/12/2021–31/07/2023)	1826 (61.4)	1 146 970 (61.5)

Abbreviations: BMI, body mass index; CNS, central nervous system; COPD, chronic obstructive airway disease; COVID-19, coronavirus disease 2019; CVD, cardiovascular disease; GP, general practice; HRT, hormone replacement therapy; IMD, Index of Multiples Deprivation; NSAID, nonsteroidal anti-inflammatory drug; PCC, post–COVID-19 condition; PCI, percutaneous transluminal coronary intervention; SARs-CoV-2, severe acute respiratory syndrome coronavirus 2; SD, standard deviation; SMD, standardized mean difference.

^a^SMD indicates difference in mean or proportion of covariates in the treatment vs nontreatment group divided by the pooled SD. SMD < 0.2 indicates a negligible difference in covariates between both groups.

^b^The frequency of hormonal replacement therapy used in the past 180 days before baseline was calculated based on female participants only.

^c^CNS drugs use includes all primary care prescriptions from British National Formulary chapter 4.9 drugs used in Parkinsonism and related disorders and 4.11 drugs for dementia.

^d^CVD drugs use includes all primary care prescriptions from British National Formulary chapters 2.1 drugs positive inotropic drugs, 2.2 diuretics, 2.3 antiarrhythmia drugs, 2.4 beta-adrenoreceptor blocking drugs, 2.5.1 vasodilator antihypertensive drugs, 2.6 nitrates, calcium-channel blockers & other antianginal drugs, 2.7.2 sympathomimetics and other vasoconstrictor drugs, 2.8 anticoagulants, 2.9 antiplatelet, 2.11 antifibrinolytic drugs, and 2.12 lipid-regulating drugs.

**Figure 2.**
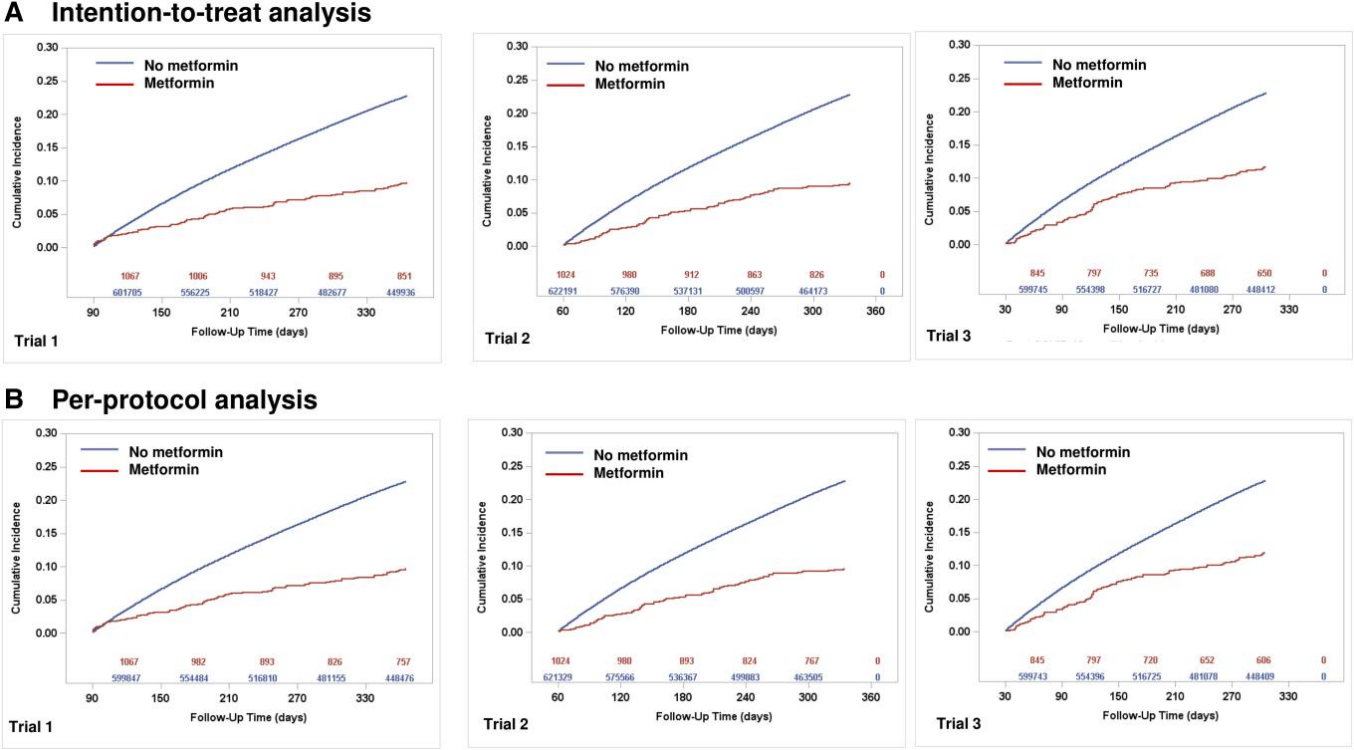
Cumulative incidence curves of PCC events among patients with and without metformin therapy during follow-up (observational analogue to an intention- to-treat [*A*] and per-protocol [*B*] analysis), 2020–2023. Abbreviations: COVID-19, coronavirus disease 2019; PCC, post–COVID-19 condition.

In emulated trials, 288 individuals among individuals who initiated metformin developed PCC events during the follow-up period, whereas 413 686 individuals among individuals who did not initiate metformin non-metformin group experienced PCC events. The estimates of PCC events comparing metformin users with non-metformin users under each target trial, along with the pooled estimate across all trials, are summarized in Table 2, and the cumulative incidence curves for each emulated trial are shown in Figure 2*A*-*B*. The estimated observational analogue of the ITT RD for PCC events at 1 year was −12.58% (95% CI, −13.77 to −11.58) with HR 0.36 (95% CI, 0.32–0.41). Estimates for PCC were similar in the per-protocol analysis, with an RD of −12.74% (95% CI, −13.89% to −11.76) and HR = 0.36 (95% CI, 0.33-0.41).

**Table 2.** Estimated Incidence Rate per 100-person Year, Risk Differences at 1 y, and Hazard Ratios for PCC Events Comparing Metformin Treatment to no Metformin Treatment

…	Treatment	PCC Events	Total Person Years	Risk Difference At 1 y (%) (95% CI)	Adjusted Hazard Ratio (95% CI)
Intention-to-treat analysis					
Pooled estimate across all trials	No metformin	413 686	1 500 521	Reference	Reference
	Metformin	288	2482	−12.58 (−13.77 to −11.58)	0.36 (0.32–0.41)
Target Trial 1	No metformin	137 992	552 173	Reference	Reference
	Metformin	100	993	−12.88 (−14.60 to −11.15)	0.38 (0.31– 0.47)
Target Trial 2	No metformin	137 899	500 098	Reference	Reference
	Metformin	92	851	−13.30 (−15.06 to −11.54)	0.34 (0.28–0.42)
Target Trial 3	No metformin	137 795	448 250	Reference	Reference
…	Metformin	96	638	−11.25 (−13.42 to −9.07)	0.39 (0.31– 0.48)
Per-protocol analysis					
Pooled estimate across all trials	No metformin	413 402	1 498 501	Reference	Reference
	Metformin	283	2416	−12.74 (−13.89 to −11.76)	0.36 (0.33– 0.41)
Target Trial 1	No metformin	137 804	550 759	Reference	Reference
	Metformin	96	960	−13.21 (−14.90 to −11.52)	0.38 (0.31– 0.46)
Target Trial 2	No metformin	137 803	499 493	Reference	Reference
	Metformin	91	831	−13.38 (−15.13 to −11.63)	0.35 (0.28– 0.43)
Target Trial 3	No metformin	137 795	448 249	Reference	Reference
…	Metformin	96	625	−11.25 (−13.42 to −9.07)	0.39 (0.32– 0.49)

Abbreviations: CI, confidence interval; COVID-19, coronavirus disease 2019; PCC, post–COVID-19 condition.

All results from the sensitivity and subgroup analyses are summarized in Tables 3 and 4. In the subgroup analyses, stratified by age, gender, presence of diabetes mellitus status, BMI category, the dominant SARS-CoV-2 variants at the time of COVID-19 diagnosis, and BMI distribution across individuals with diabetes and nondiabetes, we found that the effect of metformin in reducing the incidence of PCC was consistent across all subgroups. A forest plot illustrating results from the subgroup analyses can be found in Supplementary Fig. 6. Results from the subgroup analyses in each individual emulated trial is in Supplementary Table 5. In the sensitivity analyses, the results from the complete case analysis were consistent with the main analysis, yielding an HR of 0.35 (95% CI, 0.30–0.41). Additionally, the negative control outcome analysis, which used cancer events occurring between 90 and 365 days after the COVID-19 diagnosis as the outcome, produced an HR of 1.13 (95% CI, 0.61–1.77), further supporting the robustness of our findings. These sensitivity analyses were also conducted in each emulated trial and the results are in Supplementary Tables 6 and 7. Last, when the analysis was replicated using a traditional cohort design, the HR was 0.34 (95% CI, 0.30–0.38) (Supplementary Table 8).

**Table 3.** Subgroup Analysis: Risk of Developing PCC in the Metformin Treatment Group Comparing to no Metformin Treatment Group

Outcome	Treatment	PCC Events	Total Person Years	Adjusted Hazard Ratio (95% CI)
Diabetic status	…	…	…	…
Nondiabetes	No metformin	402 974	1 463 654	Reference
	Metformin	138	1165	0.33 (0.28–0.39)
Diabetes	No metformin	10 712	36 867	Reference
	Metformin	150	1316	0.39 (0.34–0.48)
Gender				
Male	No metformin	125 331	604 380	Reference
	Metformin	119	1176	0.36 (0.32–0.41)
Female	No metformin	288 355	896 141	Reference
	Metformin	169	1305	0.38 (0.31–0.47)
Age				
< 45 y	No metformin	169 160	579 019	Reference
	Metformin	61	681	0.23 (0.17–0.29)
≥ 45 y	No metformin	244 526	921 503	Reference
	Metformin	227	1801	0.42 (0.36–0.47)
BMI category				
BMI <30 kg/m^2^ (overweight)	No metformin	222 351	855 691	Reference
	Metformin	90	832	0.34 (0.2–0.41)
BMI ≥30 kg/m^2^ (obese)	No metformin	191 335	644 830	Reference
	Metformin	198	1650	0.39 (0.3–0.42)
Dominant SAR-CoV-2 variants at the COVID-19 diagnosis date				
Pre-Alpha and Alpha period (≤17/05/2021)	No metformin	43 855	141 163	Reference
	Metformin	71	407	0.52 (0.41–0.68)
Delta period (18/05/2021 to 13/12/2021)	No metformin	112 969	452 497	Reference
	Metformin	59	604	0.33 (0.25–0.41)
Omicron period (14/12/2021 to 31/07/2023)	No metformin	256 862	906 861	Reference
	Metformin	158	1471	0.32 (0.27–0.38)
Individuals with diabetes who are overweight or obese				
Overweight	No metformin	4310	14 945	Reference
	Metformin	44	7822	0.38 (0.28–0.50)
Obese	No metformin	6402	21 922	Reference
	Metformin	106	919	0.42 (0.34–0.51)
Individuals with nondiabetes who are overweight or obese				
Overweight	No metformin	218 041	1 046 210	Reference
	Metformin	46	543	0.30 (0.23–0.39)
Obese	No metformin	184 933	622 908	Reference
	Metformin	92	730	0.36 (0.29–0.37)

Abbreviations: BMI, body mass index; CI, confidence interval; COVID-19, coronavirus disease 2019; PCC, post–COVID-19 condition; SARS-CoV-2, severe acute respiratory syndrome coronavirus 2.

**Table 4.** Results of the Sensitivity Analyses: Risk of Developing PCC in the Complete Case Analysis and Negative Control Outcome (Cancer) Analysis

Outcome	Metformin		No Metformin		Adjusted Hazard Ratio (95% CI)
	No. of Events	Total person-years	No. of Events	Total person-years	
Post-COVID-19 condition	148	1320	228 202	815 670	0.35 (0.3–0.41)
Cancer	16	4267	6466	2 764 087	1.1 (0.61–1.77)

Abbreviations: CI, confidence interval; COVID-19, coronavirus disease 2019; PCC, post–COVID-19 condition.

## DISCUSSION

In this study using a target trial emulation framework, we found that individuals with overweight or obesity who initiated metformin within 3 months after a COVID-19 diagnosis had a lower 1-year risk of developing PCC compared to those who did not initiate metformin. The absolute RD ranged from −11.25% to −13.30%, indicating a substantial reduction in PCC risk.

### Comparison With Other Studies

Our finding aligns with the finding from the recent COVID-OUT trial, which reported that metformin reduced the risk of PCC by 63% for those starting metformin within 4 days of COVID-19 diagnosis date [23]. The COVID-OUT trial restricted metformin use to within 14 days following a COVID-19 diagnosis. Our current study expanded that restriction to allow participants to initiate metformin within 90 days after COVID-19 diagnosis and continue its use until they developed PCC, died, or reached the end of the follow-up period. The cumulative incidence of PPC in the COVID-OUT trial by day 300 was 6.3% in the metformin group, wheres it was notably higher in our study at 11.6%. This discrepancy may be attributed the differences in how PCC was defined. In the COVID-OUT trial, the diagnosis of PCC was defined as being made by a medical provider. In contrast, our study defined PCC based on the presence of either a PCC diagnostic code using Read Code in CPRD Aurum or the occurrence of at least one of 25 WHO-listed symptoms between 90 and 365 days after COVID-19 diagnosis, with the condition that none of these symptoms was present 180 days before SARS-CoV-2 infection. Additionally, there are important demographic differences between the 2 studies: the majority of the participants in our study caught SARS-CoV-2 infection during the Omicron-dominant variant period, whereas the Delta variant was predominant during the COVID-OUT trial. These factors likely contributed to differences in the results, particularly in the subgroup analysis.

A recent cohort study found that prevalent use of metformin in type 2 diabetes patients was linked to a reduced risk of PCC and mortality 6 months after SARS-CoV-2 infection [31]. PCC was defined by either a U09.9 diagnosis code within 180 days or a computable phenotype of 25 conditions in the 30–180 days postinfection. Data from 2 databases yielded different results: the National COVID Cohort Collaborative showed significant risk reductions (HR = 0.79; 95% CI, 0.71–0.88 and HR = 0.85; 95% CI, 0.78–0.92), whereas the National Patient-Centered Clinical Research Network showed no significant reductions (HR = 0.87; 95% CI, 0.66–1.14 and HR = 1.04; 95% CI, 0.97–1.11). However, these results are not directly comparable to our study, as we excluded prevalent metformin users, used a different PCC definition timeframe, had a longer follow-up period, and included patients regardless of their diabetes status, making our findings more clinically relevant.

Some studies have suggested that antivirals such as nirmatrelvir/ritonavir, molnupiravir, and remdesivir taken during the acute infection period may reduce the risk of select PCC symptoms [32–34]. However, those antivirals are not widely used in the United Kingdom because they are limited to treating COVID-19 in patients and patients at risk of severe SARS-CoV-2 infection [35, 36]. Metformin is considered cost-effective, safe, well-tolerated, and associated with a very low risk of hypoglycemia, with a long history of use in treating diabetes and polycystic ovary syndrome [37]. Previous studies have shown that metformin can reduce SARS-CoV-2 viral load [16, 38, 39]. The possible mechanism could be the suppression of protein translation via targeting the mammalian target of rapamycin pathway [16]. However, the exact mechanism of action remains unclear. Further research is needed to understand the underlying mechanisms of metformin on PCC, which could provide valuable insights into how metformin prevents PCC and might mitigate PCC symptoms.

### Strengths and Limitations of This Study

As we considered that individuals included in our study may meet an eligibility criteria at many times, this motivated us to use a sequential trial emulation framework that has been recognized to effectively reduce the bias in observational study [40]. Moreover, our study is strengthened by a large sample size, and we also included participants aged 18 years and older in our study, which enhances the generalizability of our findings and provides a more comprehensive understanding of metformin's potential in preventing PCC across a wider population.

Our study had several potential limitations. First, an International Classification of Diseases, 10th revision, code for PCC was introduced for clinical use in October 2021. Consequently, relying on this code would not capture PCC cases that occurred before this date, potentially leading to an underestimation of cases [41]. To mitigate this limitation, we adopted an alternative definition of PCC from a previous study [26], which used 25 WHO-listed symptoms as our main PCC definition. However, in the absence of a universally accepted definition of PCC and given the broad and nonspecific nature of its symptoms [42], it remains challenging to distinguish between sequelae directly attributable to COVID-19 and those arising from unrelated conditions. These inherent complexities highlight the need for rigorously designed, placebo-controlled interventional studies to more definitively assess causality. Second, given the retrospective, observational nature of the study, metformin initiators were more likely than noninitiators to have either on-label or off-label indications for metformin use, such as diabetes. Metformin initiators and noninitiators are systematically different in the baseline characteristics as we observed. To address these baseline differences, we applied propensity score fine stratification weighting, which achieved good balance between the groups. Furthermore, we used a target trial emulation framework to minimize common biases in observational studies such as prevalent user bias. However, the possibility of residual confounding cannot be entirely excluded. Third, the dosage and formulation of metformin was not taken into consideration. Fourth, this study faced limitations in statistical power for certain subgroups (eg, COVID-19 vaccination status), which restricted our ability to draw a conclusion from subgroup analysis results. Fifth, because our study focused exclusively on individuals with overweight or obesity, the findings may not be generalizable to patients with a normal BMI. Last, further research is needed to determine whether these results apply to a broader population.

### Conclusions

In summary, our findings support the protective effect of metformin treatment within 90 days of SARS-COV-2 infection on developing PCC in overweight/obesity patients in real-world settings. These findings emphasize the role of using metformin in early COVID-19 treatment plans for overweight/obesity individuals to help lower the risk of developing PCC. Further randomized controlled trials are needed to confirm the causal relationship between metformin use and its efficacy in treating PCC in overweight/obesity individuals.

## Supplementary Material

ciaf429_Supplementary_Data

## Supplementary Data

Supplementary materials are available at *Clinical Infectious Diseases* online. Consisting of data provided by the authors to benefit the reader, the posted materials are not copyedited and are the sole responsibility of the authors, so questions or comments should be addressed to the corresponding author.
