# Supplementary material for: Effect of Metformin on the Risk of Post-COVID-19 Condition Among Individuals With Overweight or Obese: A Population-based Retrospective Cohort Study
Source: Clin Infect Dis. 2025 Sep 1;82(3):e423–32. doi: 10.1093/cid/ciaf429 (PMC13016790; doi:10.1093/cid/ciaf429)
Supplement: ciaf429_Supplementary_Data [file ciaf429_supplementary_data.docx]

**Supplementary Appendix to:**

**Effect of metformin on the risk of post-COVID-19 condition among individuals with overweight and obese: a population-based retrospective cohort study**

Ubonphan Chaichana, MSc^1^; Kenneth KC Man, PhD^1,2,3,4^; Chengsheng Ju, PhD^1,5^; Janine Makaronidis, PhD^6,7,8^, Prof Li Wei, PhD^1,2,3^

^1^Research Department of Practice and Policy, UCL School of Pharmacy, London, United Kingdom

^2^Laboratory of Data Discovery for Health (D24H), Hong Kong Science Park, Hong Kong Special Administrative Region, China

^3^Centre for Medicines Optimisation Research and Education, University College London Hospitals National Health Service (NHS) Foundation Trust, 250 Euston Rd, London NW1 2PG UK

^4^Centre for Safe Medication Practice and Research, Department of Pharmacology and Pharmacy, Li Ka Shing Faculty of Medicine, The University of Hong Kong, Hong Kong Special Administrative Region, China

^5^Institute of Cardiovascular Science, University College London, London, UK

^6^Centre for Obesity Research, Rayne Institute, Department of Medicine, University College London, London, UK.

^7^National Institute of Health and Care Research, University College London Hospitals Biomedical Research Centre, London, UK.

^8^Department of Diabetes and Metabolism, Royal London Hospital, Barts Health NHS Trust, London, UK

Corresponding author

Prof Li Wei ([l.wei@ucl.ac.uk](mailto:l.wei@ucl.ac.uk))

UCL School of Pharmacy, 29-39 Brunswick Square, London, WC1N 1AX UK

**Table of Contents**

[Supplementary Table 1: Brief protocol specification and emulation of the pragmatic target of metformin and the risk of developing PCC using data from the CPRD Aurum 3](#_Toc194061819)

[Supplementary Fig.1: Study design diagram 5](#_Toc194061820)

[Supplementary Fig. 2: Illustration of sequential trial emulation for study for metformin therapy 6](#_Toc194061821)

[Supplementary Methods 1: Outcome definition 7](#_Toc194061822)

[Supplementary Table 2: Baseline characteristics of the trial 1 study cohort before emulating target trials of metformin therapy and the risk of developing PCC event, both before and after propensity-score-based fine stratification 8](#_Toc194061823)

[Supplementary Fig.3: Unweighted and weighted propensity score (PS) distribution for baseline characteristics of the trial 1 study cohort 13](#_Toc194061824)

[Supplementary Table 3: Baseline characteristics of the trial 2 study cohort before emulating target trials of metformin therapy and the risk of developing PCC event, both before and after propensity-score-based fine stratification 14](#_Toc194061825)

[Supplementary Fig.4: Unweighted and weighted propensity score (PS) distribution for baseline characteristics of the trial 2 study cohort 19](#_Toc194061826)

[Supplementary Table 4: Baseline characteristics of the trial 3 study cohort before emulating target trials of metformin therapy and the risk of developing PCC event, both before and after propensity-score-based fine stratification 20](#_Toc194061827)

[Supplementary Fig.5: Unweighted and weighted propensity score (PS) distribution for baseline characteristics of the trial 3 study cohort 25](#_Toc194061828)

[Supplementary Fig. 6: Hazard ratios (HR) and 95% confidence intervals (CI) from sensitivity analyses of metformin treatment versus no metformin treatment on PCC. 26](#_Toc194061829)

[Supplementary Table 5: Subgroup analyses of 3 emulated trials by baseline gender, age, BMI, presence of diabetes status, COVID-19 dominant variant at time of COVID-19 diagnosis 27](#_Toc194061830)

[Supplementary Table 6: Sensitivity analysis adjusting for missing data using a Complete Case Analysis (CCA) 29](#_Toc194061831)

[Supplementary Table 7: Sensitivity analysis of using cancer diagnosis between 90 to 365 days after COVID-19 diagnosis date as a negative control outcome 30](#_Toc194061832)

[Supplementary Table 8: Sensitivity analysis of using traditional cohort study design 31](#_Toc194061833)

[References 32](#_Toc194061834)

# **Supplementary Table 1: Brief protocol specification and emulation of the pragmatic target of metformin and the risk of developing PCC using data from the CPRD Aurum**

| **Component** | **Target trial Specification** | **Target Trial Emulation** |
| --- | --- | --- |
| **Eligibility criteria** | - Age ≥ 18 at study enrolment  - Have a recent history (within 60 days) of confirmed positive SARs-CoV2-infection record between 1^st^ March 2020 and 31^st^ March 2023 in CPRD Aurum (ascertained using ICD-10 code and READ codes)  - Have body mass index (BMI) ≥ 25 kg/m^2^  - Individuals require at least one year of post-registration data in CPRD Aurum database with data linkage for the Hospital Episode Statistics (HES) and The Office for National Statistics (ONS) to ensure adequate recording of medical history  - No metformin contraindication (hepatic or renal failure or lactic acidosis*)  - No prescription for metformin and any other glucose-lowering drug within 1 year  - No history of using nirmatrelvir or nirmatrelvir/ritonavir when they had SARs-CoV-2 infection | Same as for the target trial  We additionally excluded patients without 1-year record history in the database. |
| **Treatment strategies** | Metformin vs no metformin   1. Initiation of metformin within 30 days from a study index date and continuation over follow-up (1 year after a COVID-19 diagnosis date) or until development of Post-COVID-19 condition (PCC) symptom/PCC diagnosis or development of metformin contraindication (hepatic or renal failure or lactic acidosis) or die or end of follow-up period 2. No initiation of metformin over the follow-up period | Same as for the target trial  We defined the date of metformin initiation to be the first date of a prescription. We used 120 days prescription gap as a cut off day for metformin discontinuation. |
| **Treatment allocation** | Randomisation without blinding. Individuals will be randomly assigned to one of the two strategies. | Individuals are assigned to the treatment arm that they are compatible with at the baseline. |
| **Outcomes** | PCC diagnosis (Read codes or ICD-10) between 90-365 days after COVID-19 diagnosis or PCC symptoms in patients’ electronic health record defined as having at least one of 25 WHO-listed symptoms between 90-365 days after the COVID-19 diagnosis, with no history of these symptoms 180 days before SARS-CoV-2 infection | Same as for the target trial |
| **Follow-up** | Follow-up started from the study index date until the occurrence of the outcome of PCC/PCC symptoms, death, loss to follow-up (transfer out of the practice or incomplete follow-up), death, end of the study period (31^st^ July 2023), or 1 year after COVID-19 diagnosis date, whichever occurs first. | Same as for the target trial |
| **Causal contrast / estimand** | Intention-to-treat (ITT) effect  Per protocol effect | Observational analogue of the ITT effect.  Observational analogue of the per-protocol; individuals will be censored if they deviate from their assigned treatment strategy. |
| **Statistical analysis** | ITT analysis: Cox proportional hazard models with an indicator for assigned treatment strategies.  Per-protocol analysis: Cox-proportional hazard models to fit the data after censoring individuals when they deviate from their assigned treatment.  Subgroup analysis by gender, BMI (overweight versus obese), ages at baseline (<45 versus ≥45 years), presence of diabetes status, and dominant SARS-CoV-2 variants at the COVID-19 diagnosis date (pre-Alpha/Alpha versus Delta versus Omicron). | Same as for target trial with sequential emulation and adjustment for baseline covariates using propensity-score fine stratification (ATE)  Sensitivity analysis: A negative control outcome analysis using cancer diagnosis occurring between 90 to 365 days after the COVID-19 and a complete-case analysis by excluding all observations with missing data. |

*Hepatic impairment is defined using a diagnostic code for hepatic failure or ALT≥ 120 IU/L; Renal impairment is defined using a diagnostic code for renal failure, end-stage renal disease or eGFR < 30mL/min/1.73m^2^; Lactic acidosis is defined using a diagnostic code for lactic acidosis

# **Supplementary Fig.1: Study design diagram**


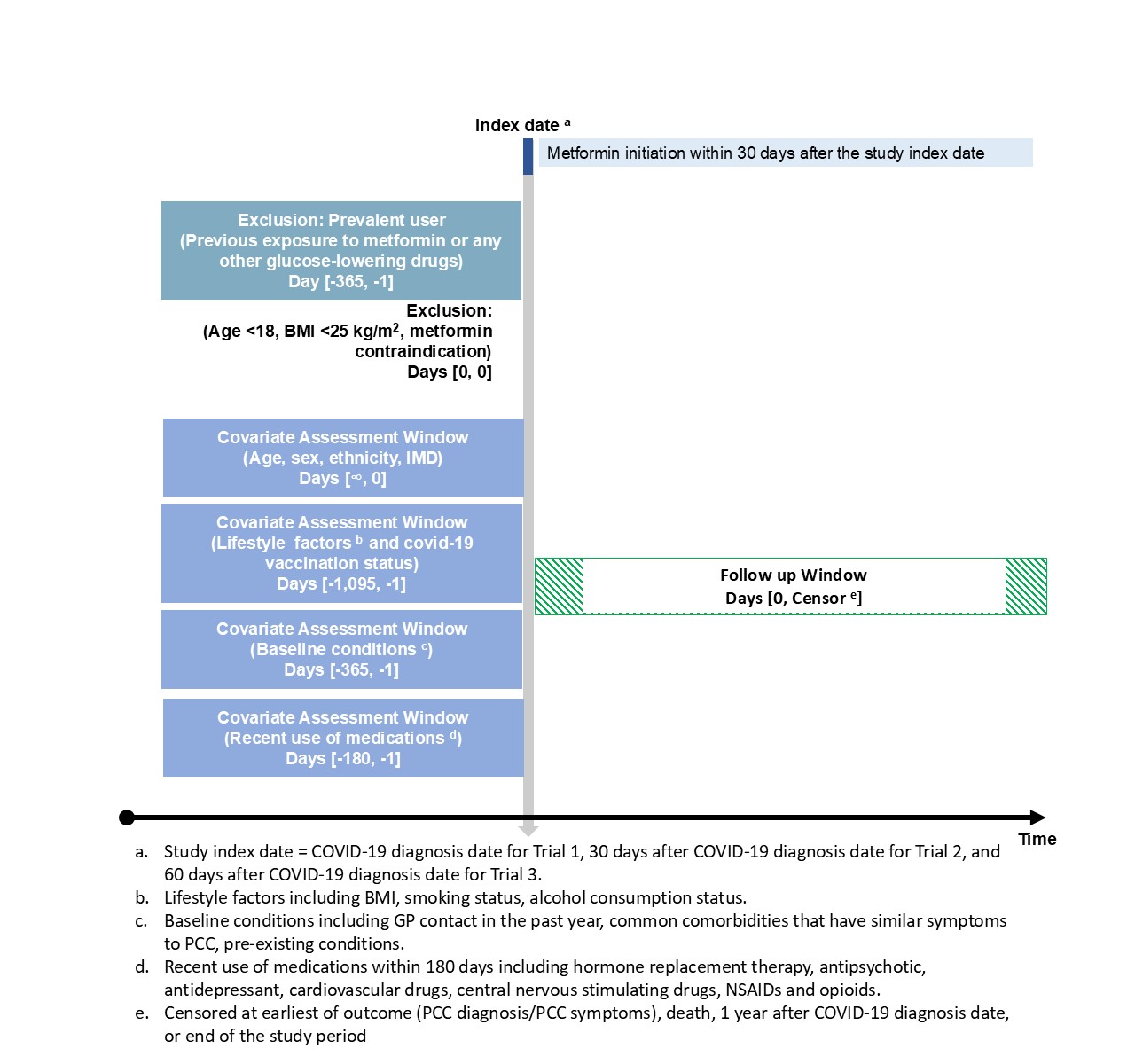


# **Supplementary Fig. 2: Illustration of sequential trial emulation for study for metformin therapy**

**
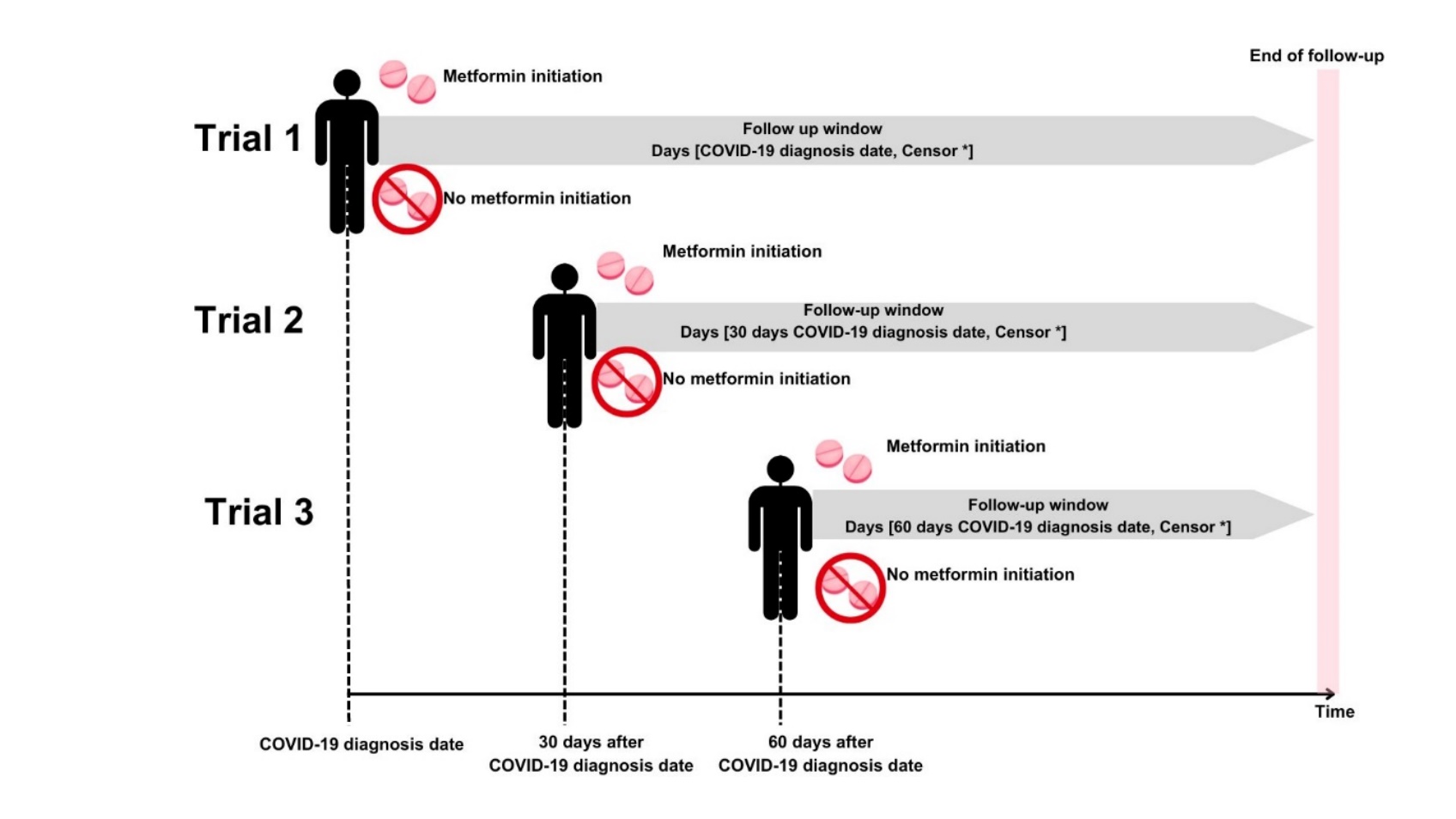
**

Notes: A sequence of target trials was emulated using electronic health record from the Clinical Practice Research Datalink (CPRD) Aurum between 1^st^ March 2020 and 31^st^ March 2023 To investigate the effect of early initiation of metformin therapy, we emulated three target trials in total, applying the same eligibility criteria in each trial. For the treatment arm, only new users with a new record of metformin prescription within 30 days after each index date were included in the trial. The individuals contributed to the treatment arm were no longer eligible for a subsequent trial. However, the individuals who did not initiate metformin can be eligible for subsequent trials. The data from these 3 emulated trials were pooled into one single model using individual-level meta-analysis for hazard ratio and risk difference at 1 year with 500 samples nonparametric bootstrapping for calculating 95% confidence interval. Propensity- score fine stratification (ATE) was employed to adjust for baseline covariates. Individuals were followed-up until the occurrence of the outcome of PCC/PCC symptoms, death, loss to follow-up (transfer out of the practice or incomplete follow-up), death, end of the study period (31^st^ July 2023), or 1 year after COVID-19 diagnosis date, whichever occurs first

# **Supplementary Methods 1: Outcome definition**

In our study, we defined post-COVID-19 condition (PCC) using SNOMED-CT or ICD-10-CM diagnostic codes for PCC in CPRD Aurum between 90-365 days after COVID-19 diagnosis date. Additionally, we also used a record of having at least one of 25 WHO-listed symptoms between 90-365 days after the COVID-19 diagnosis, with no history of these symptoms 180 days before SARS-CoV-2 infection as PCC symptoms in our study.

PCC diagnostic codes and 25 WHO-listed symptoms for PCC are presented below here.

|  | **ICD-10-CM code** | **SNOMED-CT code** |
| --- | --- | --- |
| **Diagnostic code**^(1)^ |  |  |
| Post-COVID-19 syndrome | U09.9 | 1325161000000102 |
| Ongoing symptomatic disease caused by severe acute respiratory syndrome coronavirus 2 |  | 1325161000000106 |

**25 WHO-listed symptoms for PCC including^(2)^:**

1. Abdominal pain
2. Allergy
3. Altered smell or taste
4. Anxiety
5. Brain fog
6. Blurred vision
7. Chest pain or angina
8. Cough
9. Depression without recurrence
10. Dizziness
11. Dyspnoea
12. Fatigue and malaise
13. Gastrointestinal issues
14. Headache
15. Intermittent fever
16. Joint pain
17. Memory issues
18. Menstrual problems
19. Muscle spasms and pain
20. Neuralgia
21. Pins and needles sensation
22. Post exertional fatigue
23. Sleep disorder
24. Tachycardia
25. Tinnitus and hearing problems

# **Supplementary Table 2: Baseline characteristics of the trial 1 study cohort before emulating target trials of metformin therapy and the risk of developing PCC event, both before and after propensity-score-based fine stratification**

**(Trial 1: Index date = COVID-19 diagnosis date)**

|  | **Before PS fine stratification** | | | **After PS fine stratification** | | |
| --- | --- | --- | --- | --- | --- | --- |
|  | **Metformin initiators**  **(n=1,090)** | **Non-initiators**  **(n=623,218)** | **SMD*** | **Metformin initiators**  **(n=1,087)** | **Non-initiators**  **(n=607,961)** | **SMD*** |
| **Age(years), mean(SD)** | 55.32 (15.8) | 49.55 (15.4) | 0.37 | 55.31 (15.8) | 55.26 (16.3) | 0.003 |
| **BMI, kg/m^2^, n (%)** | | | | | | |
| Overweight  (25-29.9) | 315 (28.9) | 321,744 (51.6) | -0.4764 | 314 (28.9) | 167,318 (27.5) | 0.030 |
| Obesity class I  (30-34.9) | 357 (32.6) | 177,033 (28.4) | 0.094 | 357 (32.8) | 200,531 (32.9) | -0.003 |
| Obesity class II  (35-39.9) | 226 (20.7) | 75,274 (12.1) | 0.235 | 225 (20.7) | 129,695 (21.3) | -0.016 |
| Obesity class III (≥40) | 192 (17.6) | 49,167 (7.9) | 0.294 | 191 (17.6) | 110,417 (18.2) | -0.015 |
| **Sex, n (%)** | | | | | | |
| Male | 545 (50.0) | 243,476 (39.1) | 0.221 | 542 (49.9) | 302,398 (49.7) | 0.002 |
| Female | 545 (50.0) | 379,742 (60.9) | -0.221 | 545 (50.1) | 305,563 (50.3) | -0.002 |
| **Ethnicity, n (%)** | | | | | | |
| White British | 792 (72.7) | 498,107 (79.9) | -0.171 | 789 (72.6) | 437,492 (72.0) | -0.001 |
| Asian | 98 (9.0) | 35,957 (5.8) | 0.123 | 98 (9.0) | 55,926 (9.2) | -0.006 |
| Black | 65 (6.0) | 17,733 (2.9) | 0.152 | 65 (6.0) | 37,264 (6.1) | -0.006 |
| Others | 43 (3.9) | 19,595 (3.14) | 0.043 | 43 (3.96) | 24,546 (4.0) | -0.004 |
| Mixed | 10 (0.9) | 6,235 (1.0) | -0.009 | 10 (0.9) | 5,780 (1.0) | -0.003 |
| Missing | 82 (7.5) | 45,591 (7.3) | 0.008 | 82 (7.5) | 46,954 (7.7) | -0.007 |
| **Smoking status, n (%)** | | | | | | |
| Non-smoker | 581 (53.3) | 355,898 (57.1) | -0.077 | 580 (53.3) | 323,888 (53.3) | 0.002 |
| Ex-smoker | 334 (30.6) | 167,533 (26.9) | 0.083 | 333 (30.6) | 186,238 (30.6) | 0 |
| Current smoker | 46 (4.2) | 24,447 (3.9) | 0.015 | 46 (4.2) | 26,237 (4.3) | -0.004 |
| Missing | 129 (11.8) | 75,340 (12.1) | -0.008 | 128 (11.8) | 71,598 (11.8) | 0 |
| **Alcohol consumption status, n (%)** | | | | | | |
| Current drinker | 941 (86.3) | 517,867 (83.1) | 0.9 | 938 (86.3) | 523,446 (86.1) | 0.006 |
| Non drinker | 50 (4.6) | 18,660(3.0) | 0.05 | 50 (4.6) | 28,635(4.7) | -0.024 |
| Ex-drinker | 0 (0) | 184 (0.1) | -0.243 | 0 (0) | 185 (0.1) | -0.024 |
| Missing | 99 (9.1) | 86507 (13.9) | -1.509 | 99 (9.1) | 55,715 (9.2) | -0.002 |
| **GP contact in the past year, n (%)** | | | | | | |
| 1-11 times | 210 (19.3) | 173,820 (27.9) | -0.204 | 208 (19.1) | 112,802 (18.6) | 0.015 |
| 12-18 times | 205 (18.8) | 147,112 (23.6) | -0.118 | 205 (18.9) | 113,540 (18.7) | 0.005 |
| 19-29 times | 265 (24.3) | 151,196 (24.3) | 0.001 | 265 (24.4) | 149,338 (24.6) | -0.004 |
| > 29 times | 410 (37.6) | 150,537 (24.2) | 0.295 | 409 (37.6) | 232,281 (38.2) | -0.012 |
| **Index of Multiples Deprivation (IMD), n (%)** | | | | | | |
| 1 (Least deprived) | 103 (9.5) | 81,422 (13.1) | -0.115 | 103 (9.5) | 57,175 (9.4) | 0.002 |
| 2 | 126 (11.6) | 85,118 (13.7) | -0.063 | 126 (11.6) | 68,700 (11.3) | 0.009 |
| 3 | 192 (17.6) | 111,646 (17.9) | -0.008 | 190 (17.5) | 106,179 (17.5) | 0.001 |
| 4 | 253 (23.2) | 117,430 (18.8) | 0.107 | 253 (23.3) | 142,572 (23.5) | -0.004 |
| 5 (Most deprived) | 249 (22.8) | 132,269 (21.2) | 0.039 | 249 (22.9) | 139,887 (23.0) | -0.002 |
| Missing | 167 (15.3) | 95,333 (15.3) | 0.001 | 166 (15.3) | 93,448 (15.4) | -0.003 |
| **Comorbidities, n (%)** | | | | | | |
| COPD | 55 (5.1) | 16,492 (2.7) | 0.125 | 55 (5.1) | 29,657 (4.9) | 0.008 |
| Asthma | 244 (22.4) | 144,769 (23.2) | 0.02 | 244 (22.5) | 136,618 (22.5) | -0.001 |
| Fibromyalgia | 31 (2.8) | 12,808 (2.1) | 0.051 | 31 (2.9) | 18,027 (3.0) | -0.007 |
| Anxiety | 256 (23.5) | 159,067 (25.5) | -0.047 | 256 (23.6) | 144,199 (23.7) | -0.004 |
| Depression | 148 (13.6) | 77,450 (12.4) | 0.034 | 148 (13.6) | 82,781 (13.6) | 0 |
| Migraine | 128 (11.7) | 90,521 (14.5) | -0.082 | 127 (11.7) | 71,824 (11.8) | -0.004 |
| Arrhythmia | 135 (12.4) | 25,100 (4.0) | 0.308 | 134 (12.3) | 72,259 (11.9) | 0.013 |
| Osteoporosis | 230 (21.1) | 88,710 (14.2) | 0.181 | 230 (21.2) | 127,562 (21.0) | 0.004 |
| Fragility | 8 (0.7) | 5,682 (0.9) | -0.019 | 8 (0.7) | 5,020 (0.8) | -0.01 |
| Eczema | 240 (22.0) | 146,934 (23.6) | -0.037 | 240 (22.1) | 134,709 (22.2) | -0.002 |
| Type 2 diabetes | 717 (65.8) | 15,585 (2.5) | 1.791 | 715 (65.8) | 402,480 (66.2) | -0.01 |
| Hypertension | 428 (39.3) | 135,272 (21.7) | 0.389 | 425 (39.1) | 240,411 (39.5) | -0.009 |
| Liver disease | 11 (1.0) | 2,910 (0.5) | 0.063 | 11 (1.0) | 5,658 (0.9) | 0.008 |
| Dementia | 8 (0.7) | 3,430 (0.6) | 0.023 | 8 (0.7) | 4,881 (0.8) | -0.008 |
| Chronic kidney disease | 91 (8.4) | 25,640 (4.1) | 0.176 | 89 (8.2) | 51,078 (8.4) | -0.008 |
| Stroke | 60 (5.5) | 16,453 (2.6) | 0.145 | 59 (5.4) | 33,615 (5.5) | -0.005 |
| Coronary heart disease | 100 (9.2) | 23,028 (3.7) | 0.225 | 99 (9.1) | 55,086 (9.1) | 0.002 |
| Heart failure | 139 (12.8) | 9,572 (1.5) | 0.446 | 137 (12.6) | 68,703 (11.3) | 0.04 |
| PCI | 20 (1.8) | 4,818 (0.8) | 0.094 | 19 (1.8) | 11,254 (1.9) | -0.008 |
| Rheumatoid | 20 (1.8) | 8,732 (1.4) | 0.034 | 20 (1.8) | 10,615 (1.8) | 0.007 |
| Peptic ulcer | 26 (2.4) | 9,555 (1.5) | 0.062 | 26 (2.4) | 14,730 (2.4) | -0.002 |
| Cancer | 75 (6.9) | 28,444 (4.6) | 0.099 | 74 (6.8) | 41,922 (6.9) | -0.003 |
| Myocardial infarction | 93 (8.5) | 12,071 (1.9) | 0.299 | 91 (8.4) | 46,835 (7.7) | 0.0246 |
| **Use of medication in the past 6 months, n (%)** | | | | | | |
| HRT† , (% of women | 20 (3.7) | 46,023 (12.1) | -0.317 | 20 (3.7) | 13156 (3.6) | 0.006 |
| Antipsychotic | 40 (3.7) | 16,267 (2.6) | 0.061 | 40 (3.7) | 23,150 (3.8) | -0.007 |
| Antidepressants | 268 (24.6) | 143,255 (23.0) | 0.038 | 268 (24.7) | 151,099 (24.9) | -0.004 |
| CNS drugs use‡ | 3 (0.3) | 1,245 (0.2) | 0.016 | 3 (0.3) | 1,999 (0.3) | -0.01 |
| CVD drugs use§ | 556 (51.0) | 189,883 (30.5) | 0.428 | 555 (51.1) | 314,919 (51.8) | -0.015 |
| NSAIDs | 387 (35.5) | 163,504 (26.2) | 0.202 | 386 (35.5) | 216,381 (35.6) | -0.002 |
| Opioid drug | 454 (41.7) | 151,885 (24.4) | 0.374 | 453 (41.7) | 255,141 (42.0) | -0.006 |
| **COVID-19 vaccination status, n (%)** | | | | | | |
| unvaccinated | 26 (2.4) | 6,349 (1.0) | 0.01 | 25 (2.3) | 15,010 (2.5) | -0.001 |
| 1 dose | 38 (3.5) | 25,096 (4.0) | -0.028 | 38 (3.5) | 21,315 (3.5) | -0.001 |
| 2 doses | 267 (24.5) | 207,515 (33.3) | -0.195 | 267 (24.6) | 145,383 (23.9) | 0.015 |
| 3 doses or more | 477 (43.8) | 287,322 (46.1) | -0.047 | 476 (43.8) | 267,182 (44.0) | -0.003 |
| Missing | 282 (25.9) | 97,036 (15.6) | 0.256 | 281 (25.6) | 159,071 (26.2) | -0.007 |
| **Dominant SARs-CoV-2 variants at the COVID-19 diagnosis date, n (%)** | | | | | | |
| Pre-Alpha and Alpha period (≤17/05/2021) | 225 (20.6) | 58,187 (9.3) | 0.321 | 224 (20.6) | 126,955 (20.9) | -0.007 |
| Delta period (18/05/2021 - 13/12/2021) | 238 (21.8) | 182,082 (29.2) | -0.169 | 238 (21.9) | 129,822 (21.4) | 0.013 |
| Omicron period (14/12/2021 - 31/07/2023) | 627 (57.5) | 382,949 (61.5) | -0.08 | 625 (57.5) | 351,181 (57.8) | -0.005 |

Abbreviations: SD = standard deviation; SMD = standardised mean difference; BMI = body mass index; GP = general practice; IMD= Index of Multiples Deprivation; COPD = chronic obstructive airways disease; PCI = Percutaneous transluminal Coronary Intervention; CNS = central nervous system; CVD = cardiovascular disease; NSAIDs = non-steroidal anti-inflammatory drugs

^*^SMD indicates difference in mean or proportion of covariates in the treatment vs non-treatment group divided by the pooled standard deviation. SMD of less than 0.2 indicates a negligible difference in covariates between both groups.

^†^The frequency of HRT used in the past 180 days before baseline was calculated based on female participants only.

‡CNS drugs use includes all primary care prescriptions from British National Formulary chapters 4.9 drugs used in Parkinsonism and related disorders and 4.11 drugs for dementia.

§CVD drugs use includes all primary care prescriptions from British National Formulary chapters 2.1 drugs positive inotropic drugs, 2.2 diuretics, 2.3 anti-arrhythmia drugs, 2.4 beta-adrenoreceptor blocking drugs, 2.5.1 vasodilator antihypertensive drugs, 2.6 nitrates, calcium-channel blockers & other antianginal drugs, 2.7.2 sympathomimetics and other vasoconstrictor drugs, 2.8 anticoagulants, 2.9 antiplatelet, 2.11 antifibrinolytic drugs, and 2.12 lipid-regulating drugs.

# **Supplementary Fig.3: Unweighted and weighted propensity score (PS) distribution for baseline characteristics of the trial 1 study cohort**


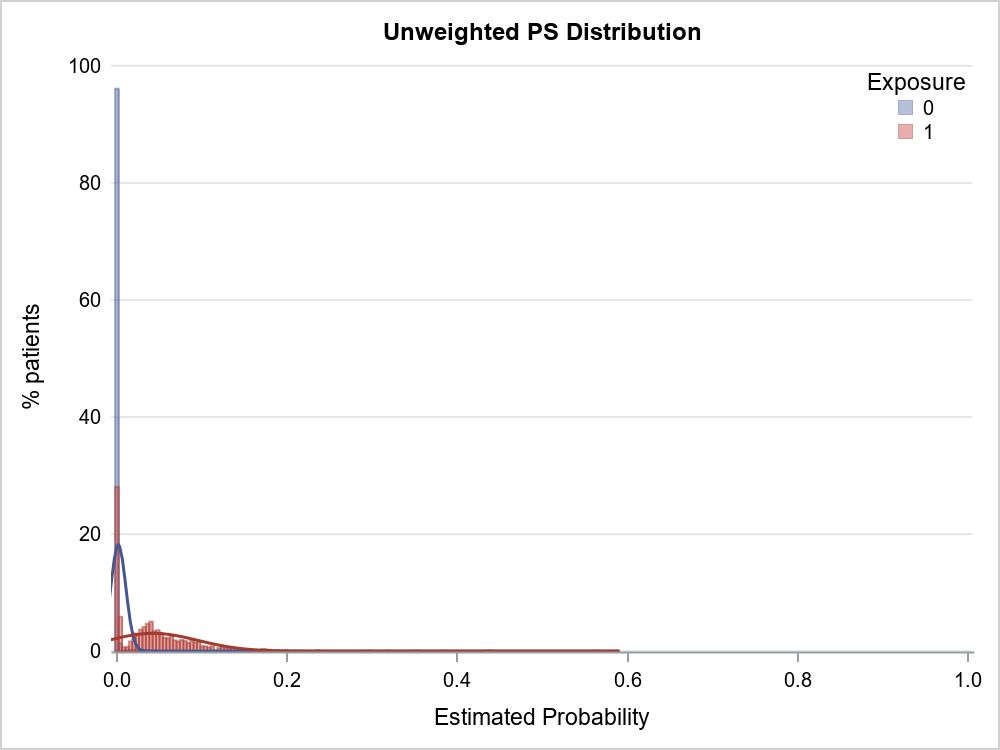

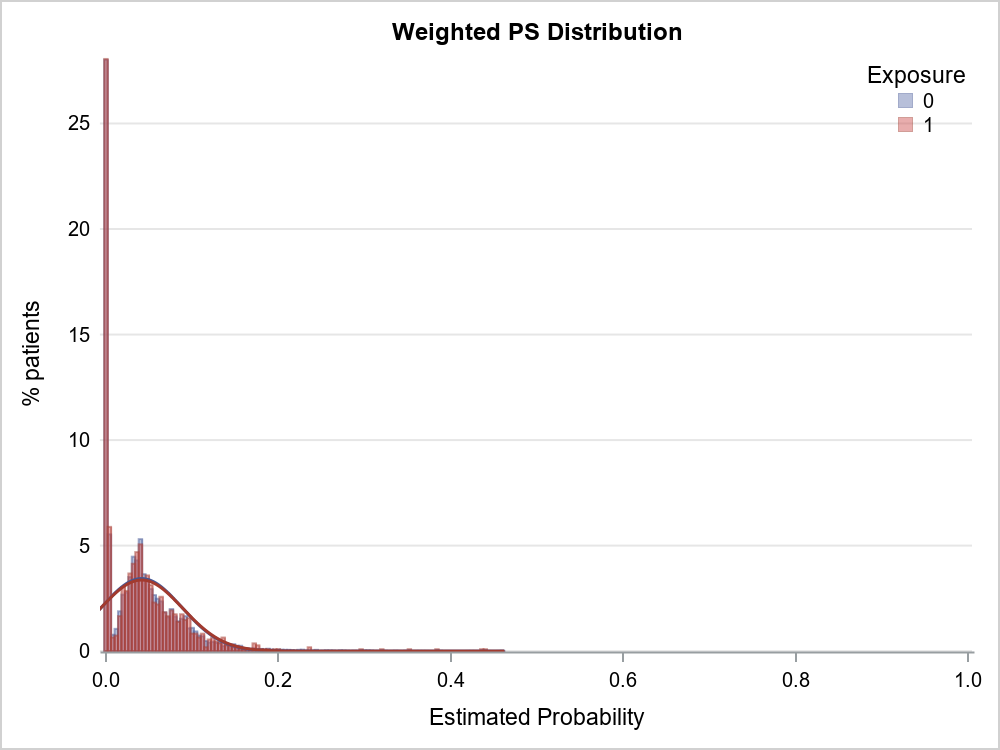


# **Supplementary Table 3: Baseline characteristics of the trial 2 study cohort before emulating target trials of metformin therapy and the risk of developing PCC event, both before and after propensity-score-based fine stratification**

**(Trial 2: Index date = 30 days after COVID-19 diagnosis date)**

|  | **Before PS fine stratification** | | | **After PS fine stratification** | | |
| --- | --- | --- | --- | --- | --- | --- |
|  | **Metformin initiators**  **(n=1,024)** | **Non-initiators**  **(n=622,192)** | **SMD*** | **Metformin initiators**  **(n=1,023)** | **Non- initiators**  **(n=605,084)** | **SMD*** |
| **Age(years), mean(SD)** | 54.8 (15.6) | 49.6 (15.4) | 0.34 | 54.8 (15.6) | 54.9 (16.2) | -0.008 |
| **BMI, kg/m^2^, n (%)** | | | | | | |
| Overweight  (25-29.9) | 297 (29.0) | 321,446 (51.7) | -0.475 | 297 (29.0) | 159,469 (26.4) | 0.059 |
| Obesity class I  (30-34.9) | 307 (30.0) | 176,726 (28.4) | 0.035 | 307 (30.0) | 185,327 (30.6) | -0.014 |
| Obesity class II  (35-39.9) | 228 (22.3) | 75,045 (12.1) | 0.271 | 228 (22.3) | 140,810 (23.3) | -0.024 |
| Obesity class III (≥40) | 192 (18.8) | 48,975 (7.9) | 0.324 | 191 (18.7) | 119,478 (19.8) | -0.027 |
| **Sex, n (%)** | | | | | | |
| Male | 494 (48.2) | 242,981 (39.1) | 0.186 | 494 (48.3) | 291,955 (48.3) | 0.001 |
| Female | 530 (51.8) | 379,211 (61.0) | -0.186 | 529 (51.7) | 313,129 (51.8) | -0.001 |
| **Ethnicity, n (%)** | | | | | | |
| White British | 737 (72.0) | 497,368 (79.9) | -0.187 | 737 (72.0) | 431,382 (71.3) | 0.017 |
| Asian | 110 (10.7) | 35,847 (5.8) | 0.182 | 110 (10.8) | 66,452 (11.0) | -0.007 |
| Black | 43 (4.2) | 17690 (2.8) | 0.074 | 42 (4.1) | 25,987 (4.3) | -0.009 |
| Others | 14 (1.4) | 6,221 (1.0) | 0.034 | 44 (4.3) | 26,683 (4.4) | -0.005 |
| Mixed | 44 (4.3) | 19,551 (3.1) | 0.061 | 14 (1.4) | 8,860 (1.5) | -0.008 |
| Missing | 76 (7.4) | 45,515 (7.3) | 0.004 | 76 (7.4) | 45,720 (7.6) | -0.005 |
| **Smoking status, n (%)** | | | | | | |
| Non-smoker | 522 (51.0) | 35,5376 (57.1) | -0.123 | 521 (50.9) | 305,634 (50.5) | 0.008 |
| Ex-smoker | 324 (31.6) | 167,208 (26.9) | 0.105 | 324 (31.7) | 192,367 (31.8) | -0.003 |
| Current smoker | 37 (3.6) | 24,410 (3.9) | -0.016 | 37 (3.6) | 21,739 (3.6) | 0.001 |
| Missing | 141 (13.8) | 75,198 (12.1) | 0.05 | 141 (13.8) | 85,343 (14.1) | -0.009 |
| **Alcohol consumption status, n (%)** | | | | | | |
| Current drinker | 895 (87.4) | 516,970 (83.1) | 0.122 | 894 (87.4) | 530,926 (87.8) | -0.001 |
| Non drinker | 44 (4.3) | 18,616 (3.0) | 0.065 | 44 (4.3) | 26,819 (4.4) | -0.002 |
| Ex-drinker | 1 (0.1) | 183 (0.0) | -0.027 | 1 (0.1) | 555 (0.1) | 0.002 |
| Missing | 84 (8.2) | 86,423 (13.9) | -0.182 | 84 (8.2) | 46,784 (7.7) | 0.002 |
| **GP contact in the past year, n (%)** | | | | | | |
| 1-11 times | 152 (14.8) | 173,668 (27.9) | -0.323 | 152 (14.9) | 80,291 (13.3) | 0.046 |
| 12-18 times | 211 (20.6) | 146,901 (23.6) | -0.073 | 211 (20.6) | 125,188 (20.7) | -0.002 |
| 19-29 times | 275 (26.9) | 150,921 (24.3) | 0.057 | 275 (26.9) | 165,966 (27.4) | -0.001 |
| > 29 times | 385 (37.6) | 150,150 (24.1) | 0.295 | 384 (37.5) | 232,935 (38.5) | -0.002 |
| **Index of Multiples Deprivation (IMD), n (%)** | | | | | | |
| 1 (Least deprived) | 131 (12.8) | 81,291 (13.1) | -0.008 | 131 (12.8) | 76,151 (12.6) | 0.007 |
| 2 | 117 (11.4) | 85,000 (13.7) | -0.068 | 117 (11.4) | 66,741 (11.0) | 0.001 |
| 3 | 187 (18.3) | 111,458 (17.9) | 0.009 | 187 (18.3) | 110,973 (18.3) | -0.002 |
| 4 | 205 (20.0) | 117,225 (18.8) | 0.03 | 205 (20.0) | 123,069 (20.3) | -0.008 |
| 5 (Most deprived) | 228 (22.3) | 132,041 (21.2) | 0.025 | 227 (22.2) | 135,824 (22.5) | -0.006 |
| Missing | 156 (15.2) | 95177 (15.3) | -0.002 | 156 (15.3) | 92,326 (15.3) | -0.0003 |
| **Comorbidities, n (%)** | | | | | | |
| COPD | 56 (5.47) | 16,436 (2.64) | 0.144 | 56 (5.47) | 31,675 (5.23) | 0.011 |
| Asthma | 261 (25.49) | 144,507 (23.23) | 0.005 | 261 (25.51) | 154,895 (25.6) | -0.002 |
| Fibromyalgia | 26 (2.5) | 12,782 (2.1) | 0.003 | 26 (2.5) | 16,227 (2.7) | -0.009 |
| Anxiety | 262 (25.6) | 158,805 (25.5) | 0.001 | 262 (25.6) | 157,328 (26.0) | -0.009 |
| Depression | 148 (14.5) | 77,302 (12.4) | 0.06 | 148 (14.5) | 89,263 (14.8) | -0.008 |
| Migraine | 153 (14.9) | 90,368 (14.5) | 0.01 | 152 (14.9) | 93,087 (15.4) | -0.015 |
| Arrhythmia | 89 (8.7) | 25,010 (4.0) | 0.192 | 89 (8.7) | 51,163 (8.5) | 0.009 |
| Osteoporosis | 203 (19.8) | 88,505 (14.2) | 0.149 | 202 (19.8) | 120,895 (20.0) | -0.006 |
| Fragility | 18 (1.8) | 5,664 (0.9) | 0.074 | 18 (1.8) | 10,800 (1.8) | -0.002 |
| Eczema | 233 (22.8) | 146,700 (23.6) | -0.02 | 233 (22.8) | 136,797 (22.6) | 0.004 |
| Type 2 diabetes | 438 (42.8) | 15,147 (2.4) | 1.1 | 437 (42.7) | 254,478 (42.1) | 0.01 |
| Hypertension | 408 (39.8) | 134,862 (21.7) | 0.402 | 407 (39.8) | 247,979 (41.0) | -0.024 |
| Liver disease | 7 (0.7) | 2,903 (0.5) | 0.029 | 7 (0.7) | 4,254 (0.7) | -0.002 |
| Dementia | 12 (1.2) | 3,418 (0.6) | 0.067 | 12 (1.2) | 7,091 (1.2) | - |
| Chronic kidney disease | 102 (10.0) | 22,925 (3.7) | 0.251 | 79 (7.7) | 46,898 (7.8) | -0.001 |
| Stroke | 65 (6.35) | 16,388 (2.63) | 0.18 | 65 (6.35) | 37,828 (6.25) | 0.004 |
| Coronary heart disease | 102 (10.0) | 22,925 (3.7) | 0.251 | 102 (10.0) | 58,069 (9.6) | 0.013 |
| Heart failure | 110 (10.7) | 9,461 (1.5) | 0.392 | 109 (10.7) | 54,837 (9.1) | 0.053 |
| PCI | 26 (2.5) | 4,792 (0.8) | 0.139 | 26 (2.5) | 14,698 (2.4) | 0.007 |
| Rheumatoid | 13 (1.3) | 8,718 (1.4) | -0.011 | 13 (1.3) | 7,537 (1.3) | 0.002 |
| Peptic ulcer | 33 (3.2) | 9,522 (1.5) | 0.111 | 33 (3.2) | 18,644 (3.1) | 0.008 |
| Cancer | 71 (6.9) | 28,373 (4.6) | 0.102 | 71 (6.9) | 42,668 (7.1) | -0.004 |
| Myocardial infarction | 78 (7.6) | 11,993 (1.9) | 0.269 | 77 (7.5) | 42,063 (7.0) | 0.01 |
| **Use of medication in the past 6 months, n (%)** | | | | | | |
| HRT^†^ , (% of women | 32 (6.0) | 45,962 (12.1) | -0.213 | 32 (6.1) | 21,047 (5.6) | 0.019 |
| Antipsychotic | 35 (3.4) | 16,232 (2.6) | 0.047 | 35 (3.4) | 21,446 (3.5) | -0.007 |
| Antidepressants | 268 (26.2) | 142,985 (23.0) | 0.074 | 267 (26.1) | 162,026 (26.8) | -0.015 |
| CNS drugs use‡ | 1 (0.1) | 1,244 (0.2) | -0.027 | 1 (0.1) | 536 (0.1) | 0.003 |
| CVD drugs use§ | 554 (54.1) | 189,327 (30.4) | 0.494 | 553 (54.1) | 337,694 (55.8) | -0.035 |
| NSAIDs | 379 (37.0) | 163,123 (26.2) | 0.234 | 378 (37.0) | 227,670 (37.6) | -0.014 |
| Opioid drug | 457 (44.6) | 151,426 (24.3) | 0.437 | 456 (44.6) | 276,897 (45.8) | -0.024 |
| **COVID-19 vaccination status, n (%)** | | | | | | |
| unvaccinated | 20 (2.0) | 6,229 (1.0) | 0.079 | 20 (2.0) | 12,122 (2.0) | - |
| 1 dose | 27 (2.6) | 25,069 (4.0) | -0.078 | 27 (2.6) | 15,046 (2.5) | 0.01 |
| 2 doses | 277 (27.1) | 207,238 (33.3) | -0.137 | 277 (27.1) | 161,000 (26.6) | 0.011 |
| 3 doses or more | 486 (47.5) | 286,835 (46.1) | 0.027 | 486 (47.5) | 288,285 (47.6) | -0.003 |
| Missing | 214 (20.9) | 96,821 (15.6) | 0.139 | 213 (20.8) | 128,631 (21.3) | -0.011 |
| **Dominant SARs-CoV-2 variants at the COVID-19 diagnosis date, n (%)** | | | | | | |
| Pre-Alpha and Alpha period (≤17/05/2021) | 162 (15.8) | 58,025 (9.3) | 0.197 | 162 (15.8) | 96,497 (16.0) | -0.003 |
| Delta period (18/05/2021 - 13/12/2021) | 249 (24.3) | 181,833 (29.2) | -0.11 | 248 (24.2) | 144,972 (24.0) | 0.007 |
| Omicron period (14/12/2021 - 31/07/2023) | 613 (59.9) | 382,334 (61.5) | -0.033 | 613 (59.9) | 363,615 (60.1) | -0.004 |

Abbreviations: SD = standard deviation; SMD = standardised mean difference; BMI = body mass index; GP = general practice; IMD= Index of Multiples Deprivation; COPD = chronic obstructive airways disease; PCI = Percutaneous transluminal Coronary Intervention; CNS = central nervous system; CVD = cardiovascular disease; NSAIDs = non-steroidal anti-inflammatory drugs

^*^SMD indicates difference in mean or proportion of covariates in the treatment vs non-treatment group divided by the pooled standard deviation. SMD of less than 0.2 indicates a negligible difference in covariates between both groups.

^†^The frequency of HRT used in the past 180 days before baseline was calculated based on female participants only.

‡CNS drugs use includes all primary care prescriptions from British National Formulary chapters 4.9 drugs used in Parkinsonism and related disorders and 4.11 drugs for dementia.

§CVD drugs use includes all primary care prescriptions from British National Formulary chapters 2.1 drugs positive inotropic drugs, 2.2 diuretics, 2.3 anti-arrhythmia drugs, 2.4 beta-adrenoreceptor blocking drugs, 2.5.1 vasodilator antihypertensive drugs, 2.6 nitrates, calcium-channel blockers & other antianginal drugs, 2.7.2 sympathomimetics and other vasoconstrictor drugs, 2.8 anticoagulants, 2.9 antiplatelet, 2.11 antifibrinolytic drugs, and 2.12 lipid-regulating drug

# **Supplementary Fig.4: Unweighted and weighted propensity score (PS) distribution for baseline characteristics of the trial 2 study cohort**


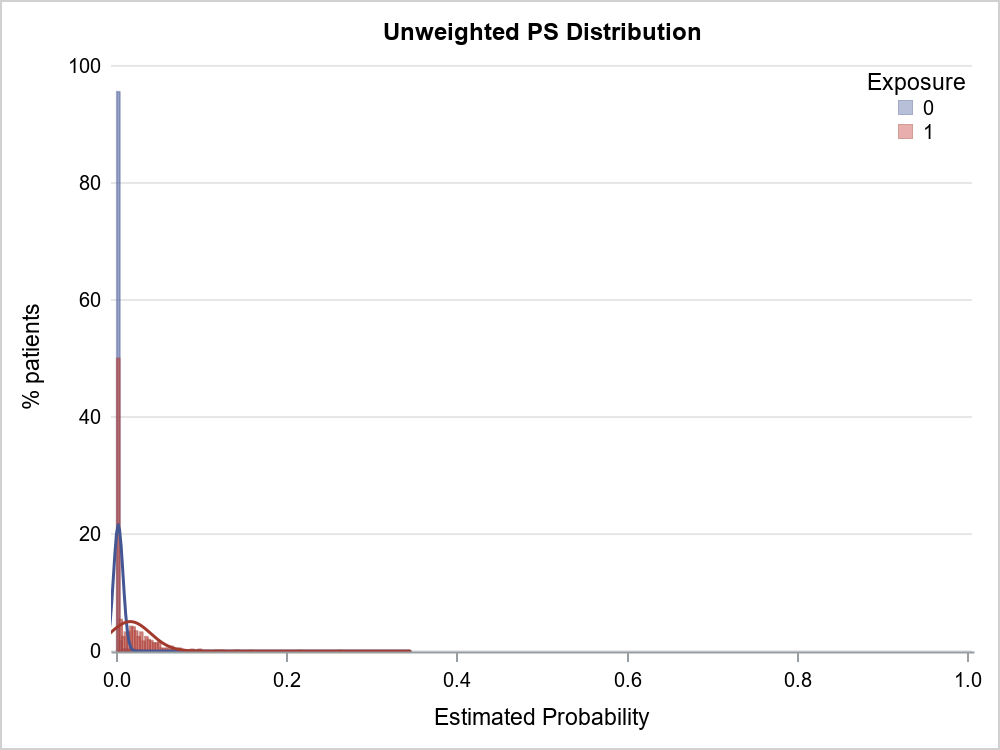

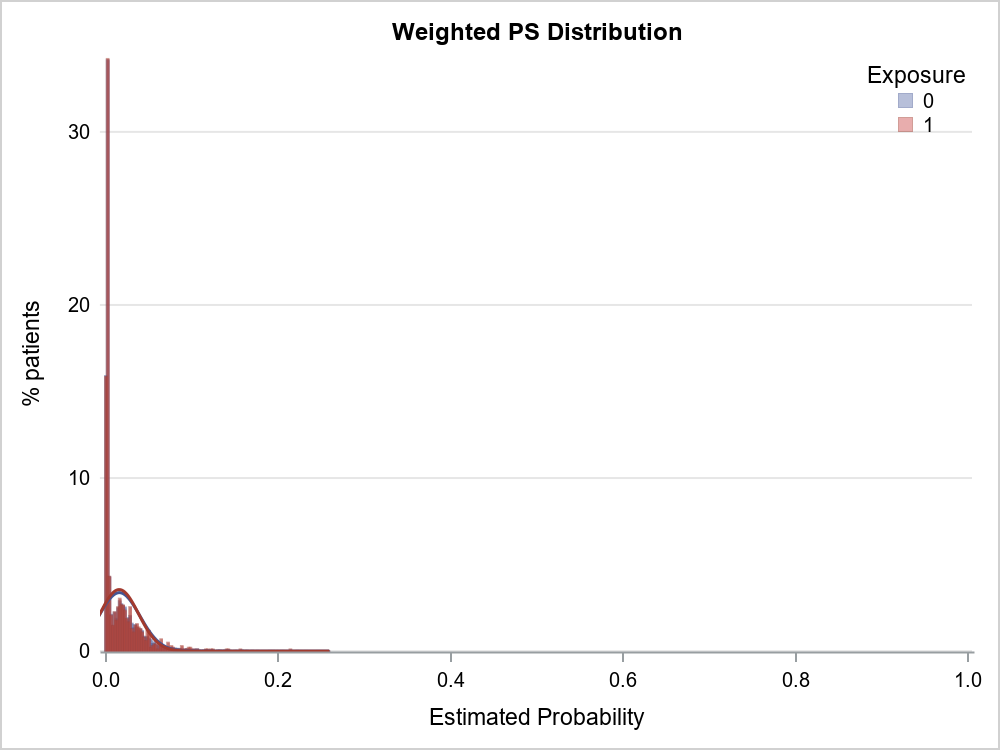


# **Supplementary Table 4: Baseline characteristics of the trial 3 study cohort before emulating target trials of metformin therapy and the risk of developing PCC event, both before and after propensity-score-based fine stratification**

**(Trial 3: Index date = 60 days after COVID-19 diagnosis date)**

|  | **Before PS fine stratification** | | | **After PS fine stratification** | | |
| --- | --- | --- | --- | --- | --- | --- |
|  | **Metformin initiators**  **(n=862)** | **Non-initiators**  **(n=621,227)** | **SMD*** | **Metformin initiators**  **(n=861)** | **Non- initiators**  **(n=603,883)** | **SMD*** |
| **Age(years), mean(SD)** | 55.2 (15.9) | 49.8 (15.4) | 0.342 | 55.2 (15.9) | 55.8 (16.8) | -0.038 |
| **BMI, kg/m^2^, n (%)** | | | | | | |
| Overweight  (25-29.9) | 416 (48.3) | 422,367 (68.0) | -0.408 | 415 (48.2) | 292,156 (48.4) | -0.004 |
| Obesity class I  (30-34.9) | 249 (28.9) | 134,767 (21.7) | 0.166 | 249 (28.9) | 175,490 (29.1) | -0.003 |
| Obesity class II  (35-39.9) | 130 (15.1) | 42,331 (6.8) | 0.267 | 130 (15.1) | 89,711 (14.9) | 0.007 |
| Obesity class III (≥40) | 67 (7.8) | 21,762 (3.5) | 0.186 | 67 (7.8) | 46,526 (7.7) | 0.003 |
| **Sex, n (%)** | | | | | | |
| Male | 366 (42.5) | 242,557 (39.0) | 0.07 | 365 (42.4) | 260,998 (43.2) | -0.017 |
| Female | 496 (57.5) | 378,670 (61.0) | -0.07 | 496 (57.6) | 342885 (56.8) | 0.017 |
| **Ethnicity, n (%)** | | | | | | |
| White British | 670 (77.7) | 496,611 (79.9) | -0.054 | 670 (77.8) | 469,100 (77.7) | 0.003 |
| Asian | 82 (9.5) | 35,760 (5.8) | 0.142 | 81 (9.4) | 56,351 (9.3) | 0.003 |
| Black | 25 (2.9) | 17,660 (2.8) | 0.003 | 25 (2.9) | 18,798 (3.1) | -0.012 |
| Others | 25 (2.9) | 19,525 (3.1) | -0.014 | 25 (2.9) | 17409 (2.9) | 0.001 |
| Mixed | 4 (0.5) | 6,217 (1.0) | -0.063 | 4 (0.5) | 2799 (0.5) | >0.001 |
| Missing | 56 (6.5) | 45,454 (7.3) | -0.032 | 56 (6.5) | 39426 (6.5) | -0.001 |
| **Smoking status, n (%)** | | | | | | |
| Non-smoker | 461 (53.5) | 354,867 (57.1) | -0.073 | 461 (53.5) | 322590 (53.2) | 0.005 |
| Ex-smoker | 267 (31.0) | 166,906 (26.9) | 0.091 | 267 (31.0) | 188,439 (31.1) | -0.002 |
| Current smoker | 40 (4.6) | 24,367 (3.9) | 0.035 | 40 (4.6) | 28,786 (4.8) | -0.005 |
| Missing | 94 (10.9) | 75,087 (12.1) | -0.037 | 94 (10.9) | 66,379 (11.0) | -0.001 |
| **Alcohol consumption status, n (%)** | | | | | | |
| Current drinker | 772 (89.6) | 522,443 (84.1) | -0.068 | 771 (89.6) | 542,622 (89.9) | -0.010 |
| Non drinker | 22 (2.6) | 17,190 (2.8) | -0.014 | 22 (2.6) | 16,232 (2.7) | -0.033 |
| Ex-drinker | 2 (0.2) | 328 (0.1) | 0.048 | 2 (0.2) | 1,484 (0.3) | -0.003 |
| Missing | 66 (7.7) | 81,266 (13.1) | -0.179 | 66 (7.7) | 43,545 (7.2) | -0.017 |
| **GP contact in the past year, n (%)** | | | | | | |
| 1-11 times | 52 (6.0) | 148,543 (23.9) | -0.518 | 52 (6.0) | 32,804 (5.4) | 0.027 |
| 12-18 times | 127 (14.7) | 148,109 (23.8) | -0.232 | 127 (14.7) | 85,656 (14.1) | 0.017 |
| 19-29 times | 238 (27.6) | 161,105 (25.9) | 0.038 | 238 (27.6) | 170,293 (28.1) | -0.011 |
| > 29 times | 445 (51.6) | 163,470 (26.3) | 0.537 | 445 (51.6) | 317,440 (52.3) | -0.015 |
| **Index of Multiples Deprivation (IMD), n (%)** | | | | | | |
| 1 (Least deprived) | 104 (12.0) | 81,174 (13.1) | -0.03 | 104 (12.1) | 71,958 (11.9) | 0.006 |
| 2 | 115 (13.3) | 84,874 (13.7) | -0.009 | 115 (13.3) | 80,336 (13.3) | 0.003 |
| 3 | 167 (19.4) | 111,264 (17.9) | 0.038 | 167 (19.4) | 116,670 (19.3) | 0.003 |
| 4 | 152 (17.6) | 117,054 (18.8) | -0.313 | 152 (17.6) | 109,363 (18.0) | -0.011 |
| 5 (Most deprived) | 200 (23.2) | 131,821 (21.2) | 0.048 | 200 (23.2) | 140,079 (23.1) | 0.002 |
| Missing | 124 (14.4) | 95,040 (15.3) | -0.026 | 124 (14.4) | 87,787 (14.5) | -0.003 |
| **Comorbidities, n (%)** | | | | | | |
| COPD | 52 (6.0) | 17,270 (2.8) | 0.159 | 52 (6.0) | 35,834 (5.9) | 0.005 |
| Asthma | 203 (23.6) | 144,452 (23.3) | 0.007 | 203 (23.6) | 140,445 (23.2) | 0.009 |
| Fibromyalgia | 29 (3.4) | 12,834 (2.1) | 0.08 | 29 (3.4) | 20,702 (3.4) | -0.003 |
| Anxiety | 260 (30.2) | 159,297 (25.6) | 0.101 | 260 (30.2) | 183,611 (30.3) | -0.003 |
| Depression | 145 (16.8) | 77,600 (12.5) | 0.123 | 145 (16.8) | 102,679 (16.9) | -0.003 |
| Migraine | 129 (15.0) | 90,466 (14.6) | 0.011 | 129 (15.0) | 91,425 (15.1) | -0.003 |
| Arrhythmia | 117 (13.6) | 25,113 (4.0) | 0.341 | 117 (13.6) | 79,424 (13.1) | -0.013 |
| Osteoporosis | 196 (22.7) | 89,289 (14.4) | 0.216 | 196 (22.7) | 141,000 (23.3) | -0.012 |
| Fragility | 9 (1.0) | 5,711 (0.9) | 0.013 | 9 (1.0) | 7,172 (1.2) | -0.013 |
| Eczema | 208 (24.1) | 146,652 (23.6) | 0.012 | 208 (24.1) | 144,868 (23.9) | 0.005 |
| Type 2 diabetes | 388 (45.0) | 16,311 (2.6) | 1.147 | 388 (45.0) | 274,359 (45.3) | -0.005 |
| Hypertension | 351 (40.7) | 135,119 (21.8) | 0.418 | 351 (40.7) | 253,860 (41.9) | -0.024 |
| Liver disease | 5 (0.6) | 2,962 (0.5) | 0.014 | 5 (0.6) | 3,916 (0.7) | -0.009 |
| Dementia | 11 (1.3) | 3,440 (0.6) | 0.076 | 11 (1.3) | 7,845 (1.3) | -0.001 |
| Chronic kidney disease | 75 (8.7) | 25,642 (4.1) | 0.188 | 75 (8.7) | 53,408 (8.8) | -0.004 |
| Stroke | 62 (7.2) | 16,406 (2.6) | 0.212 | 62 (7.2) | 42,548 (7.0) | 0.007 |
| Coronary heart disease | 84 (9.7) | 22,962 (3.7) | 0.243 | 84 (9.7) | 59,976 (9.9) | -0.005 |
| Heart failure | 125 (14.5) | 9,452 (1.5) | 0.492 | 125 (14.5) | 83,008 (13.7) | -0.023 |
| PCI | 30 (3.5) | 4,777 (0.8) | 0.189 | 30 (3.5) | 18,724 (3.1) | 0.020 |
| Rheumatoid | 20 (2.3) | 8,722 (1.4) | 0.068 | 20 (2.3) | 13,620 (2.3) | 0.005 |
| Peptic ulcer | 22 (2.6) | 9,522 (1.5) | 0.072 | 22 (2.6) | 15,700 (2.6) | -0.002 |
| Cancer | 56 (6.5) | 28,432 (4.6) | 0.084 | 56 (6.5) | 41,666 (6.9) | -0.015 |
| Myocardial infarction | 66 (7.7) | 11,983 (1.9) | 0.271 | 66 (7.7) | 43,682 (7.2) | 0.017 |
| **Use of medication in the past 6 months, n (%)** | | | | | | |
| HRT^†^ , (% of women | 28 (5.7) | 46,257 (12.2) | -0.232 | 28 (5.7) | 19,941 (5.5) | 0.007 |
| Antipsychotic | 40 (4.6) | 17,459 (2.8) | 0.097 | 40 (4.6) | 29,199 (4.8) | -0.008 |
| Antidepressants | 268 (31.1) | 143,455 (23.1) | 0.181 | 268 (31.1) | 188,928 (31.2) | -0.002 |
| CNS drugs use^‡^ | 2 (0.2) | 1288 (0.2) | 0.005 | 2 (0.2) | 1,572 (0.3) | -0.006 |
| CVD drugs use^§^ | 522 (60.6) | 192,032 (30.9) | 0.623 | 522 (60.6) | 383,820 (63.3) | -0.057 |
| NSAIDs | 368 (42.7) | 164,688 (26.5) | 0.345 | 368 (42.7) | 260,330 (42.9) | -0.005 |
| Opioid drug | 412 (47.8) | 153,189 (24.7) | 0.496 | 412 (47.8) | 302,295 (49.9) | -0.042 |
| **COVID-19 vaccination status, n (%)** | | | | | | |
| unvaccinated | 13 (1.5) | 6,370 (1.0) | 0.04 | 12 (1.5) | 7,411 (1.6) | -0.007 |
| 1 dose | 228 (26.5) | 183,291 (29.5) | -0.068 | 228 (26.4) | 158,996 (26.2) | 0.005 |
| 2 doses | 423 (49.1) | 314,955 (50.7) | -0.033 | 423 (49.1) | 297,990 (49.2) | -0.002 |
| 3 doses or more | 108 (12.5) | 42,405 (6.8) | 0.194 | 108 (12.5) | 76,221 (12.6) | -0.001 |
| Missing | 90 (10.4) | 74,206 (12.0) | -0.048 | 90 (10.4) | 63,265 (10.4) | >0.001 |
| **Dominant SARs-CoV-2 variants at the COVID-19 diagnosis date, n (%)** | | | | | | |
| Pre-Alpha and Alpha period (≤17/05/2021) | 81 (9.4) | 57,919 (9.3) | 0.002 | 81 (9.4) | 57,485 (9.5) | -0.003 |
| Delta period (18/05/2021 - 13/12/2021) | 195 (22.6) | 181,621 (29.2) | -0.151 | 195 (22.6) | 137,340 (22.6) | >0.001 |
| Omicron period (14/12/2021 - 31/07/2023) | 586 (68.0) | 381,687 (61.4) | 0.137 | 586 (68.0) | 411,367 (67.9) | 0.003 |

Abbreviations: SD = standard deviation; SMD = standardised mean difference; BMI = body mass index; GP = general practice; IMD= Index of Multiples Deprivation; COPD = chronic obstructive airways disease; PCI = Percutaneous transluminal Coronary Intervention; CNS = central nervous system; CVD = cardiovascular disease; NSAIDs = non-steroidal anti-inflammatory drugs

^*^SMD indicates difference in mean or proportion of covariates in the treatment vs non-treatment group divided by the pooled standard deviation. SMD of less than 0.2 indicates a negligible difference in covariates between both groups.

^†^The frequency of HRT used in the past 180 days before baseline was calculated based on female participants only.

^‡^CNS drugs use includes all primary care prescriptions from British National Formulary chapters 4.9 drugs used in Parkinsonism and related disorders and 4.11 drugs for dementia.

^§^CVD drugs use includes all primary care prescriptions from British National Formulary chapters 2.1 drugs positive inotropic drugs, 2.2 diuretics, 2.3 anti-arrhythmia drugs, 2.4 beta-adrenoreceptor blocking drugs, 2.5.1 vasodilator antihypertensive drugs, 2.6 nitrates, calcium-channel blockers & other antianginal drugs, 2.7.2 sympathomimetics and other vasoconstrictor drugs, 2.8 anticoagulants, 2.9 antiplatelet, 2.11 antifibrinolytic drugs, and 2.12 lipid-regulating drugs

# **Supplementary Fig.5: Unweighted and weighted propensity score (PS) distribution for baseline characteristics of the trial 3 study cohort**


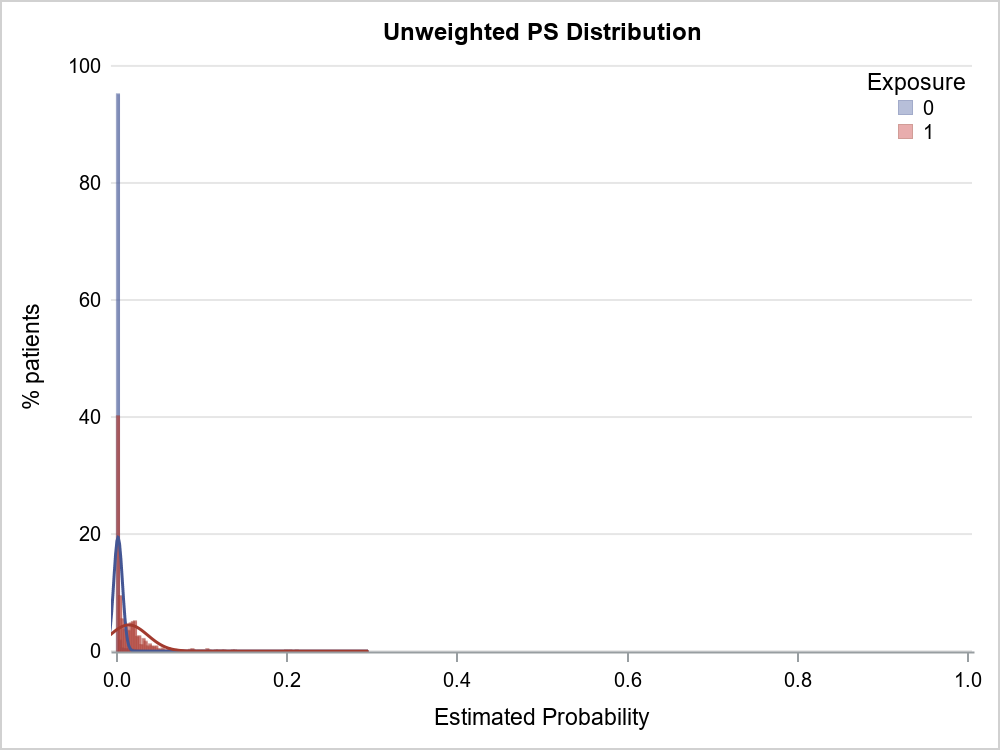

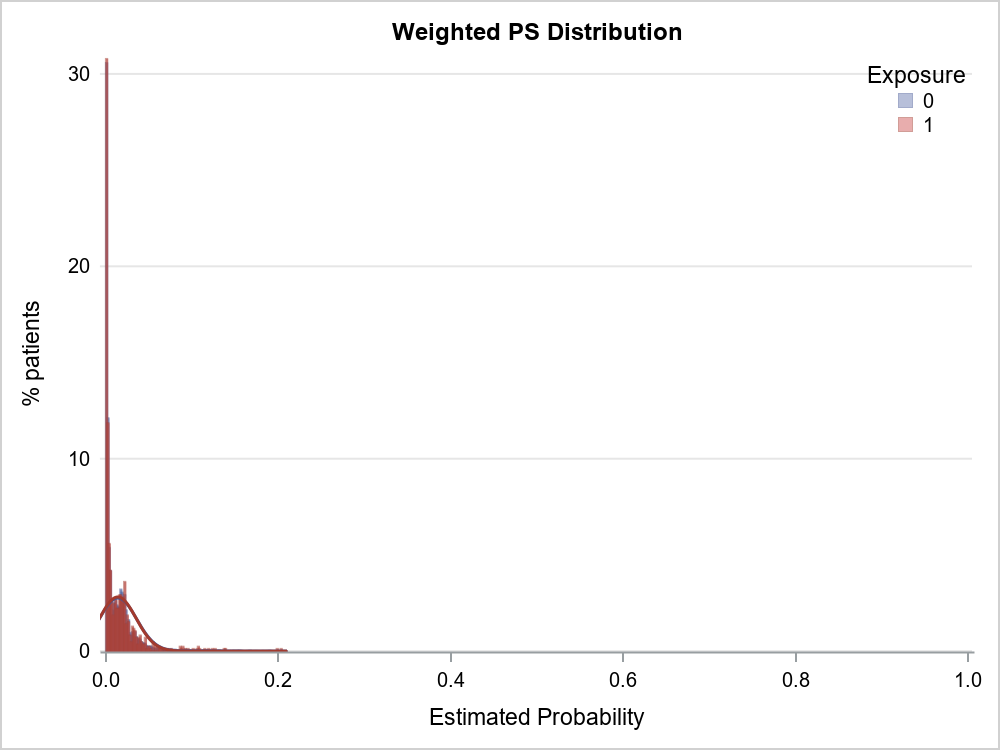


# **Supplementary Fig. 6: Hazard ratios (HR) and 95% confidence intervals (CI) from sensitivity analyses of metformin treatment versus no metformin treatment on PCC.**


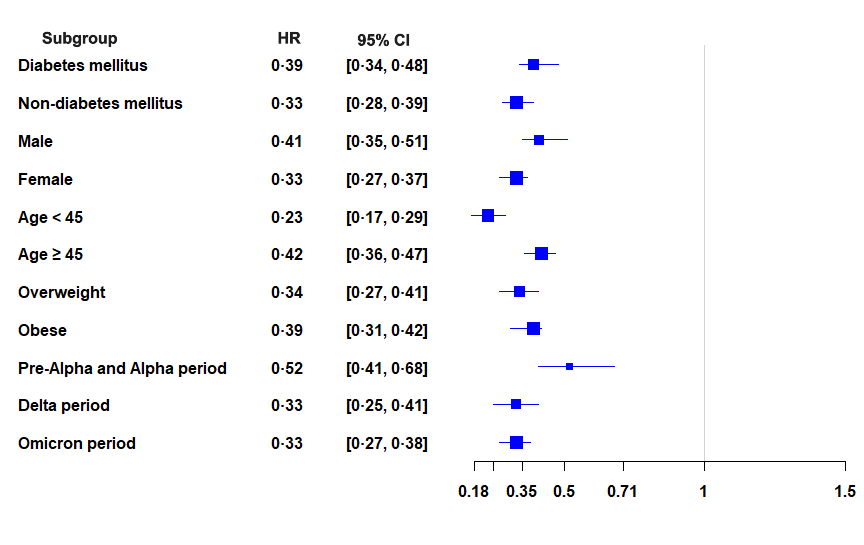


# **Supplementary Table 5: Subgroup analyses of 3 emulated trials by baseline gender, age, BMI, presence of diabetes status, COVID-19 dominant variant at time of COVID-19 diagnosis**

| **Subgroup** | **Intention-to-treat adjusted hazard ratio (95% CI)^*^** |
| --- | --- |
| **Age < 45 years** |  |
| Trial 1 (COVID-19 diagnosis date) | 0.22 (0.14 to 0.35) |
| Trial 2 (30 days after COVID-19 diagnosis date) | 0.21 (0.14 to 0.33) |
| Trial 3 (60 days after COVID-19 diagnosis date) | 0.27 (0.17 to 0.42) |
| **Age** ≥ **45 years** |  |
| Trial 1 (COVID-19 diagnosis date) | 0.45 (0.36 to 0.56) |
| Trial 2 (30 days after COVID-19 diagnosis date) | 0.41 (0.32 to 0.51) |
| Trial 3 (60 days after COVID-19 diagnosis date) | 0.44 (0.35 to 0.56) |
| **BMI 25-29.9 kg/m^2^ (Overweight)** |  |
| Trial 1 (COVID-19 diagnosis date) | 0.27 (0.17 to 0.42) |
| Trial 2 (30 days after COVID-19 diagnosis date) | 0.30 (0.20 to 0.45) |
| Trial 3 (60 days after COVID-19 diagnosis date) | 0.40 (0.30 to 0.52) |
| **BMI ≥30 kg/m^2^ (Obese)** |  |
| Trial 1 (COVID-19 diagnosis date) | 0.42 (0.34 to 0.53) |
| Trial 2 (30 days after COVID-19 diagnosis date) | 0.36 (0.29 to 0.46) |
| Trial 3 (60 days after COVID-19 diagnosis date) | 0.39 (0.29 to 0.52) |
| **Male** |  |
| Trial 1 (COVID-19 diagnosis date) | 0.39 (0.28 to 0.53) |
| Trial 2 (30 days after COVID-19 diagnosis date) | 0.42 (0.31 to 0.57) |
| Trial 3 (60 days after COVID-19 diagnosis date) | 0.46 (0.33 to 0.64) |
| **Female** |  |
| Trial 1 (COVID-19 diagnosis date) | 0.36 (0.28 to 0.47) |
| Trial 2 (30 days after COVID-19 diagnosis date) | 0.29 (0.22 to 0.38) |
| Trial 3 (60 days after COVID-19 diagnosis date) | 0.35 (0.26 to 0.45) |
| **Diabetic** |  |
| Trial 1 (COVID-19 diagnosis date) | 0.40 (0.31 to 0.52) |
| Trial 2 (30 days after COVID-19 diagnosis date) | 0.43 (0.32 to 0.58) |
| Trial 3 (60 days after COVID-19 diagnosis date) | 0.40 (0.29 to 0.56) |
| **Non diabetic** |  |
| Trial 1 (COVID-19 diagnosis date) | 0.35 (0.25 to 0.48) |
| Trial 2 (30 days after COVID-19 diagnosis date) | 0.28 (0.21 to 0.38) |
| Trial 3 (60 days after COVID-19 diagnosis date) | 0.38 (0.29 to 0.51) |
| **Pre-Alpha and Alpha period (≤17/05/2021)** |  |
| Trial 1 (COVID-19 diagnosis date) | 0.53 (0.37 to 0.78) |
| Trial 2 (30 days after COVID-19 diagnosis date) | 0.66 (0.46 to 0.94) |
| Trial 3 (60 days after COVID-19 diagnosis date) | 0.29 (0.13 to 0.65) |
| **Delta period (18/05/2021 to 13/12/2021)** |  |
| Trial 1 (COVID-19 diagnosis date) | 0.41 (0.27 to 0.62) |
| Trial 2 (30 days after COVID-19 diagnosis date) | 0.22 (0.13 to 0.37) |
| Trial 3 (60 days after COVID-19 diagnosis date) | 0.40 (0.26 to 0.62) |
| **Omicron period (14/12/2021 to 31/07/2023)** |  |
| Trial 1 (COVID-19 diagnosis date) | 0.29 (0.22 to 0.39) |
| Trial 2 (30 days after COVID-19 diagnosis date) | 0.31 (0.23 to 0.41) |
| Trial 3 (60 days after COVID-19 diagnosis date) | 0.39 (0.30 to 0.51) |

^*^Comparing metformin initiation at baseline and continuation over follow-up with no metformin initiation over follow-up.

# **Supplementary Table 6: Sensitivity analysis adjusting for missing data using a Complete Case Analysis (CCA)**

|  | **Intention-to-treat adjusted hazard ratio (95% CI)^*^** |
| --- | --- |
| **PCC diagnosis/PCC symptoms** |  |
| Trial 1 (COVID-19 diagnosis date) | 0.38 (0.28 to 0.50) |
| Trial 2 (30 days after COVID-19 diagnosis date) | 0.29 (0.21 to 0.39) |
| Trial 3 (60 days after COVID-19 diagnosis date) | 0.38 (0.29 to 0.49) |

^*^Comparing metformin initiation at baseline and continuation over follow-up with no metformin initiation over follow-up.

# **Supplementary Table 7: Sensitivity analysis of using cancer diagnosis between 90 to 365 days after COVID-19 diagnosis date as a negative control outcome**

|  | **Intention-to-treat adjusted hazard ratio (95% CI)^*^** |
| --- | --- |
| **Cancer diagnosis** |  |
| Trial 1 (COVID-19 diagnosis date) | 0.70 (0.26 to 1.88) |
| Trial 2 (30 days after COVID-19 diagnosis date) | 1.37 (0.65 to 2.91) |
| Trial 3 (60 days after COVID-19 diagnosis date) | 1.46 (0.60 to 3.53) |

^*^Comparing metformin initiation at baseline and continuation over follow-up with no metformin initiation over follow-up.

# **Supplementary Table 8: Sensitivity analysis of using traditional cohort study design**

|  | **Intention-to-treat adjusted hazard ratio (95% CI)^*^** |
| --- | --- |
| **PCC diagnosis/PCC symptoms†** | 0.34 (0.30 to 0.38) |

^*^Comparing metformin initiation at baseline and continuation over follow-up with no metformin initiation over follow-up.

^†^Maximum survival time = 275 days as the follow-up started at 90 days after COVID-19 diagnosis date.

# **References**

1. Walker AJ, MacKenna B, Inglesby P, Tomlinson L, Rentsch CT, Curtis HJ, et al. Clinical coding of long COVID in English primary care: a federated analysis of 58 million patient records in situ using OpenSAFELY. Br J Gen Pract. 2021;71(712):e806-e14.

2. Català M, Mercadé-Besora N, Kolde R, Trinh NTH, Roel E, Burn E, et al. The effectiveness of COVID-19 vaccines to prevent long COVID symptoms: staggered cohort study of data from the UK, Spain, and Estonia. Lancet Respir Med. 2024;12(3):225-36.
